# KSHV and HPV modulate epithelial-to-mesenchymal transition in oral epithelial cells

**DOI:** 10.1128/mbio.00484-25

**Published:** 2025-08-15

**Authors:** Qian Li, Sharon E. Hopcraft, Philip T. Lange, Linda Pluta, Dirk P. Dittmer, Cary A. Moody, Blossom Damania

**Affiliations:** 1 Lineberger Comprehensive Cancer Center, University of North Carolina at Chapel Hill, Chapel Hill, North Carolina, USA; 2 Department of Microbiology and Immunology, University of North Carolina at Chapel Hill 318275, Chapel Hill, North Carolina, USA; 3 Curriculum in Genetics and Molecular Biology, University of North Carolina at Chapel Hill 2331, Chapel Hill, North Carolina, USA; Massachusetts Institute of Technology, Cambridge, Massachusetts, USA; Tufts University School of Medicine, Boston, Massachusetts, USA

**Keywords:** human oncoviruses, KSHV, HPV31, oral epithelial cell, proliferation, migration, invasion, EMT, vimentin, eribulin

## Abstract

Most oral viral infections that manifest as oral diseases or cancers are caused by oncogenic human papillomavirus and herpesviruses. However, the mechanisms involved in the association between oral cancer and oncogenic virus infection are not well understood. In this study, we used telomerase (hTERT) -immortalized normal oral gingival keratinocytes (NOKs) and generated latent Kaposi’s sarcoma-associated herpesvirus (KSHV)- and human papillomavirus 31 (HPV31)-infected NOKs. NOKs either stably maintaining HPV episomes or latently infected with KSHV acquired a fibroblast-like morphology and upregulated cell proliferation in both complete media and serum starvation culture conditions. Moreover, migration and invasion assays revealed that the KSHV- and HPV-infected NOKs show markedly higher migration and invasion activity compared to uninfected cells. We demonstrated that KSHV-NOK and HPV-NOK express much lower protein levels of the epithelial cell marker E-cadherin and significantly higher protein levels of mesenchymal cell marker vimentin, indicating that KSHV and HPV31 infection induces an epithelial-to-mesenchymal transition (EMT). Notably, after efficient vimentin knockdown using two different lentiviral short hairpin RNAs (shRNAs) or eribulin treatment, E-cadherin expression was restored, and phenotypic alterations (proliferation, migration, and invasion) caused by KSHV and HPV31 infection were abolished, suggesting that KSHV- and HPV31-induced EMTs play an important role in these phenotypic alterations by KSHV and HPV31. Given that changes in EMT are often associated with cancer progression, our results offer important insight into the mechanism behind KSHV and HPV infection and cancer, suggesting that EMT is a potential therapeutic target for KSHV- and HPV-driven cancers.

**IMPORTANCE:**

The oral cavity is believed to be a primary site where many viruses infect the human body. Kaposi’s sarcoma-associated herpesvirus (KSHV) and human papillomavirus (HPV) are both found and cause cancers in the oral cavity. However, knowledge of how KSHV and HPV infection is connected to oral cancer (e.g., oncogenesis and metastasis) remains limited. Here, our study reveals that KSHV and high-risk HPV31 can induce epithelial-to-mesenchymal transition by upregulation of vimentin and downregulation of E-cadherin, which is vital for KSHV-normal oral gingival keratinocyte (NOK) and HPV-NOK to acquire cancer cell characteristics such as cell survival, migration, and invasion activities. For the first time, we show that knockdown of vimentin and eribulin treatment can restore E-cadherin and reverse epithelial-to-mesenchymal transition in KSHV- and HPV31-infected oral epithelial cells. These findings fill a gap in our understanding of oncogenesis and metastasis of oral cancers caused by KSHV and HPV31, revealing that vimentin may serve as a potential therapeutic molecular target for KSHV- and HPV-associated oral cancer.

## INTRODUCTION

The worldwide prevalence of human cancers caused by oncoviruses has been estimated to be approximately 15%–20% (1, 2). Kaposi’s sarcoma-associated herpesvirus (KSHV) and human papillomavirus (HPV) are two important human oncoviruses. KSHV is associated with human malignancies including Kaposi’s sarcoma, primary effusion lymphoma, and multicentric Castleman’s disease (3–6). Over 400 types of HPVs have been identified, and high-risk HPVs (e.g., HPV16, HPV18, HPV31, HPV45) are the etiological agents of cervical cancer and other anogenital cancers, as well as head and neck cancers (e.g., oropharyngeal cancers) (7–11). KSHV and HPV are found in the oral cavity and can cause cancers in the oral cavity (12). Oral transmission is believed to be the major transmission route of KSHV (13), since the median log titer of KSHV shed from the oral cavity is approximately 2.5 times higher than other anatomic sites (14). HPV is a sexually transmitted virus and can also be transmitted through oral sex or deep kissing, which can lead to cancers of the oropharynx (15). HPV-associated oropharyngeal cancers have increased incidence and represent an emerging viral epidemic of cancer (16). Here, we explore additional mechanisms for how KSHV and HPV contribute to oncogenesis (17, 18).

Oral epithelial cells are likely the cell type that is primarily infected with KSHV and HPV to produce new virions for subsequent infection of naïve cells and transmission to other individuals (19–21). Given the tropism of both KSHV and HPV for oral epithelial cells, and the fact that primary oral epithelial cells grow slowly and have a limited life span in *in vitro* culture conditions, we utilized hTERT-immortalized normal oral keratinocytes (NOKs) originally isolated from gingival tissue (22).

Epithelial cells can transdifferentiate into mesenchymal cells through a process called epithelial-to-mesenchymal transition (EMT), which is a critical aspect of many cancers. EMT is a functional cellular transition process during which epithelial cells acquire mesenchymal phenotypes including fibroblast-like cell morphology, enhanced resistance to apoptosis, cytoskeletal reorganization, increased cell migration and invasion, and significantly induced production of extracellular matrix (ECM) components following the repression of epithelial characteristics (23, 24). In terms of molecular mechanisms, EMT is tightly regulated by multiple signaling pathways and transcription factors, which ultimately results in a loss of epithelial cell markers such as E-cadherin, occludin, claudins, and cytokeratins, while positively regulating mesenchymal cell markers such as N-cadherin, vimentin, fibronectin, and matrix metalloproteinases (MMPs) (25–27). Vimentin is an important type III intermediate filamentous protein that is expressed mainly in cells of mesenchymal origin. However, vimentin is not just a marker of mesenchymal cells. It is well established that vimentin plays a crucial role in EMT and is at the heart of EMT-mediated metastasis (28). During EMT, vimentin works as a key regulator of multiple cellular activities, including cytoskeletal reorganization, cell migration, cell adhesion, and cell invasion, and modifies DNA repair pathways to support EMT (28).

EMT is known to occur during cancer progression, and several human oncoviruses or their individual viral proteins can induce EMT, including HPV E6 or E7 proteins, the KSHV LANA protein, and Epstein-Barr virus (29–33). However, most existing oncovirus-EMT research has not focused on oral cavity cells, especially with respect to KSHV.

To investigate whether KSHV and HPV31 can induce EMT, we first generated KSHV latently infected NOK (KSHV-NOK) and NOKs that stably maintain HPV31 episomes (HPV-NOK). We found that KSHV-NOK and HPV31-NOK exhibit altered cell morphology, increased cell proliferation, and elevated cell migration and invasion. These phenotypic changes led us to investigate whether EMT is involved. We found that KSHV and HPV31 infection significantly upregulated the mesenchymal cell marker, vimentin, and reduced the epithelial cell marker, E-cadherin. Knockdown of vimentin or treatment with eribulin, an EMT inhibitor, increased E-cadherin and reduced vimentin, resulting in decreased proliferation, migration, and invasion of KSHV- and HPV-infected cells, suggesting that EMT plays an important role in these virus-driven pathways.

## RESULTS

### Generation of KSHV- and HPV-infected NOK cells

**Fig 1:**
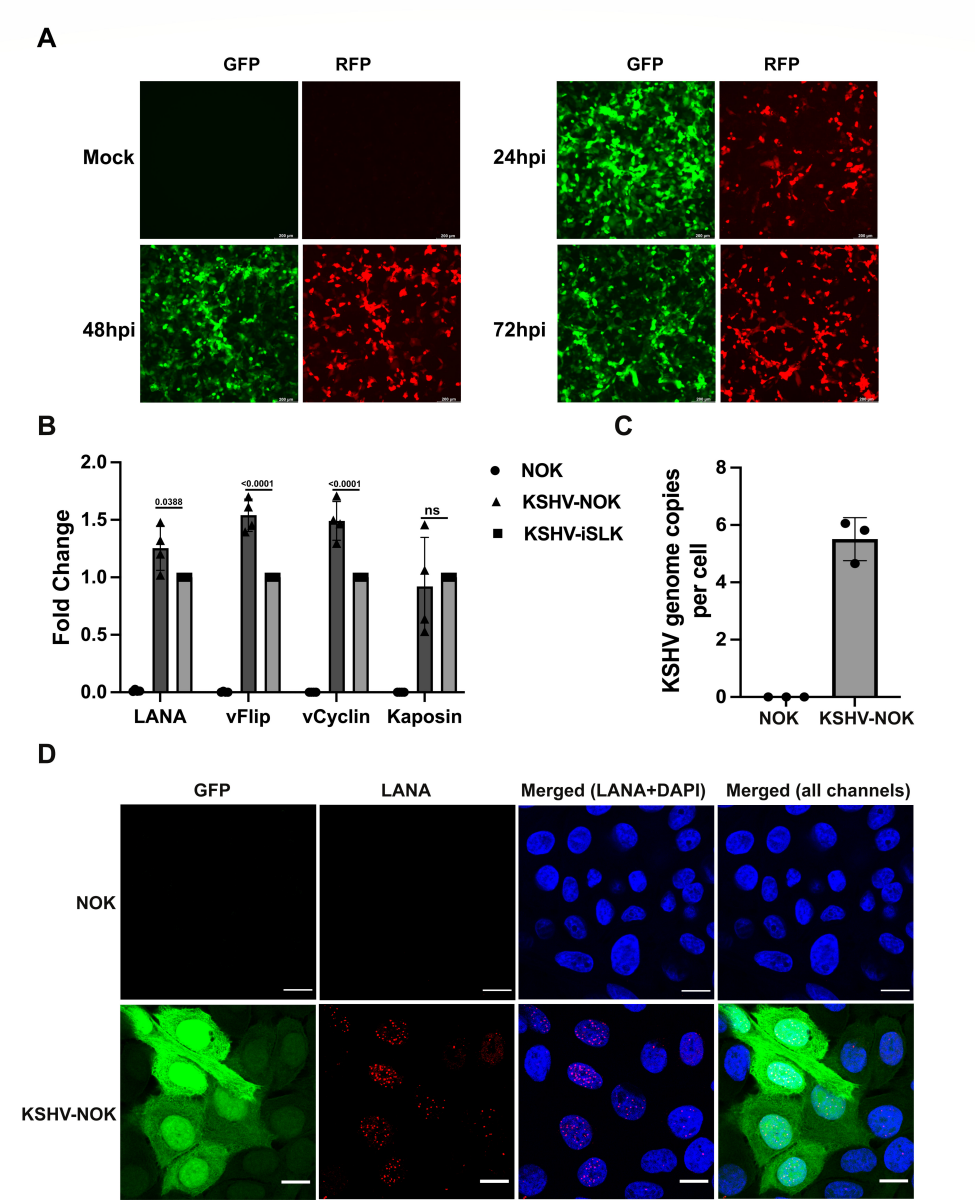
NOK supports KSHV infection and latency. (**A**) NOK cells were infected with either media (mock) or purified recombinant virus, rKSHV.219. GFP and RFP images of mock cells at 72 hpi and of rKSHV.219-infected cells at 24 hpi, 48 hpi, and 72 hpi were taken using a 10× objective of a Leica DMi8 fluorescent microscope. Scale bar, 200 µm. (**B**) Total RNA was isolated from uninfected NOK and rKSHV.219 latently infected NOK (KSHV-NOK) after puromycin selection. reverse transcription-quantitative real time polymerase chain Reaction (RT-qPCR) was performed to confirm KSHV latent gene expression at the mRNA level. mRNA isolated from rKSHV.219 latently infected iSLK (KSHV-iSLK) is shown as a positive control. (**C**) Genome copy numbers from NOK and KSHV-NOK cells were measured following 3 weeks of selection in puromycin through genomic DNA isolation and subsequent qPCR. (**D**) After at least 3 weeks of selection in puromycin, immunofluorescence was performed to validate expression of KSHV-encoded LANA as indicated by the typical intranuclear, punctate staining pattern (red dots). Nuclei were identified using 4′,6-diamidino-2-phenylindole (DAPI) (blue). Images were taken under the 63× oil objective of an LSM700 confocal microscope. Scale bar, 15 μm. (**A and D**) Images shown are representative of three independent experiments. (**B and C**) The graphs represent the average of three independent experiments, and error bars represent mean ± SEM. *P*-values in panel B were analyzed using two-way analysis of variance with Dunnett multiple comparisons.

To gain a better understanding of the KSHV life cycle and viral oncogenesis in the oral cavity, we generated a KSHV latently infected-hTERT-immortalized NOK cell line (KSHV-NOK) by *de novo* infection with purified recombinant KSHV (rKSHV.219). rKSHV.219 contains green fluorescent protein (GFP) expressed under the elongation factor-1a (EF-1a) promoter as an indicator for infection regardless of latent or lytic replication, red fluoresent protein (RFP) expressed under the KSHV lytic polyadenylated nuclear (PAN) RNA promoter during the lytic life cycle, and the gene for puromycin resistance as a selectable marker (34). The representative GFP and RFP images at different time points post *de novo* infection are shown in Fig. 1A. Almost all rKSHV.219-infected NOKs were GFP-positive at 24 h post-infection (24 hpi), while many cells were RFP-positive, indicating that lytic replication occurred. We found that the peak GFP signal is at 24 hpi, while the peak RFP signal is at 48 hpi. Cells were subjected to puromycin selection for several weeks, and KSHV latency was validated in multiple ways (35). The KSHV-NOK expressed comparable levels of KSHV latent mRNAs with the previously published cell line KSHV-inducible SLK (iSLK) (36) (Fig. 1B) and maintained approximately five to six genome copies per cell (Fig. 1C). The cells were also stained for LANA expression using an immunofluorescence assay (Fig. 1D). Confocal microscopy images revealed 100% of GFP-positive cells and LANA expression (red dots) within KSHV-NOK nuclei, while no GFP and LANA dots were observed in the uninfected NOK cells. KSHV-latently infected cells, KSHV-NOK, do not express RFP.

**Fig 2:**
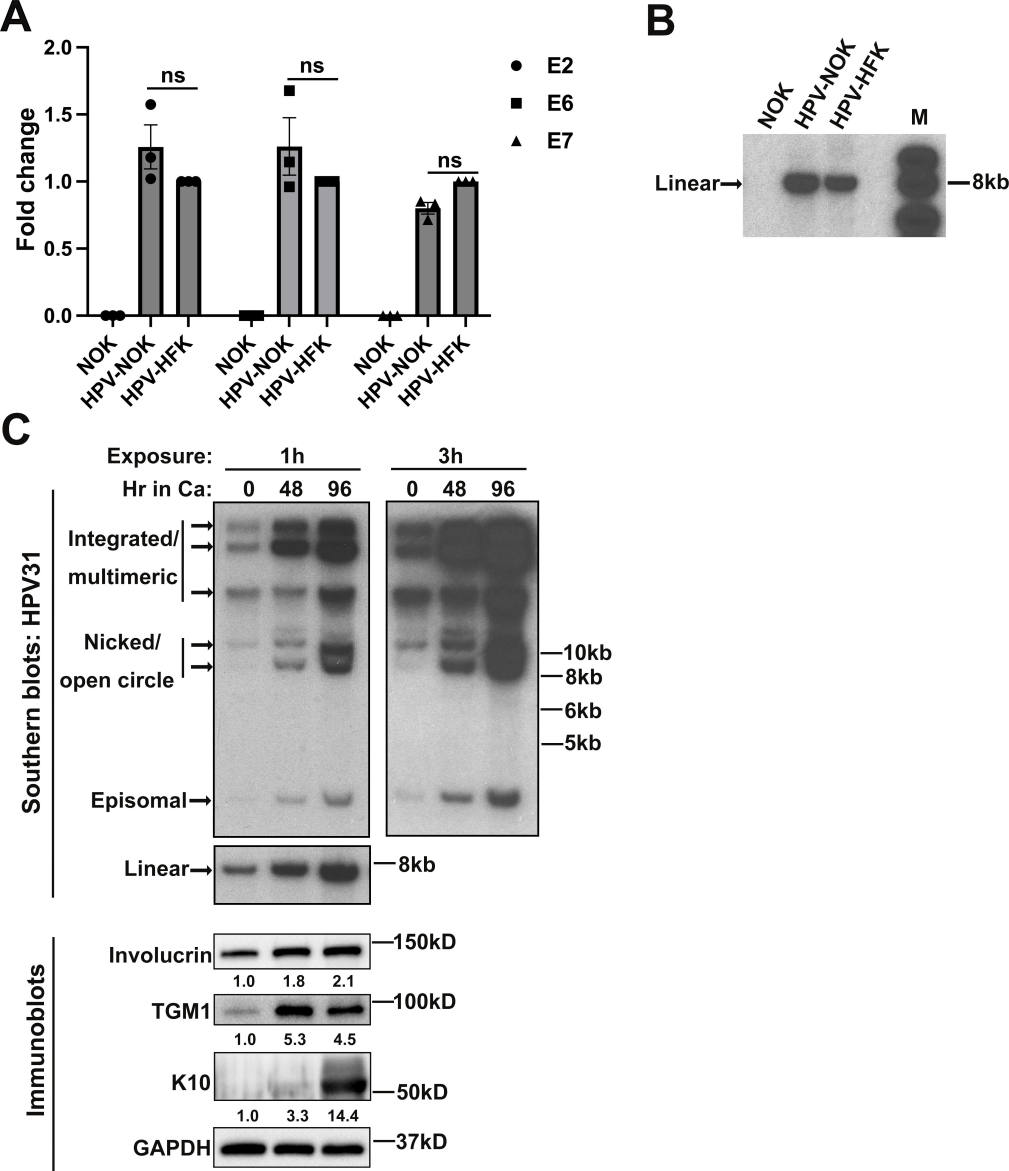
Establishment of the HPV31-positive NOK cell line. (**A**) Undifferentiated NOK, HPV-NOK, and HPV-HFK cells were harvested for total RNA isolation and RT-qPCR to confirm HPV31 early gene expression at the mRNA level. The graph represents the average of three independent experiments and error bars represent mean ± SEM. *P*-values were analyzed using two-way analysis of variance with Dunnett multiple comparisons. (**B**) Total genomic DNA was harvested from undifferentiated NOK, HPV-NOK, and HPV-HFK cells and then digested with DpnI (to remove residual input DNA) and EcoRV (to cut HPV31 DNA into linear form). Southern blot analysis on digested DNA was carried out with ^32^P-labeled HPV31 genome as the probe. Shown are representative images for four independent experiments. (**C**) Total genomic DNA and whole-cell lysate were harvested from undifferentiated HPV-NOK (0 h time point) and after differentiation in high-calcium media for 48 and 96 h. DNA was digested with DpnI + BamHI (HPV31 DNA non-cutter) or DpnI + EcoRV (cutting the HPV31 genome once). Southern blot analysis on digested DNA was carried out with a ^32^P-labeled HPV31 genome as the probe. The membranes were exposed to X-ray film for 1 h and 3 h in the −80°C freezer before exposure. Whole-cell lysate was subjected to Western blot analysis to detect differentiation markers, involucrin, transglutaminase 1 (TGM1), and keratin 10 (K10). Images shown are representative of three independent experiments. Hr, hour; Ca, calcium.

We also established an HPV31-positive NOK cell line (HPV-NOK) by co-transfecting NOK cells with the HPV31 genome (pBR322-HPV31) and a selection marker (pSV2-Neo). After 4 weeks of G418 selection, the HPV-NOK cells can persistently maintain HPV31 DNA as episomes even without selection. After 4 weeks of selection, we routinely cultured the HPV-NOK in normal media without G418. We confirmed this by performing an RT-qPCR assay to confirm that the HPV31 genome is expressed in these cells. As shown in Fig. 2A, HPV-NOK expressed a similar level of HPV31 early gene mRNAs (E2, E6, and E7) as did human foreskin keratinocytes (HFKs) stably maintaining HPV31 genomes. Southern blotting assays were then carried out to investigate whether undifferentiated HPV-NOKs express the correct size of the HPV31 linear genome. As shown in Fig. 2B, HPV-NOK expressed the correct sized HPV31 genome (around 8 kb) as HPV-HFK, while no HPV31 band was detected in the control parental NOK cells.

The HPV life cycle is tightly dependent on epithelial differentiation. To determine whether HPV-NOKs maintain HPV31 genomes episomally and that HPV31 genome amplification occurs upon differentiation, we performed HPV-NOK differentiation assays in high-calcium media (Fig. 2C). Increased protein levels of the differentiation markers, involucrin, transglutaminase 1 (TGM1), and keratin 10 (K10), indicated successful induction of differentiation. Upon differentiation, the total levels of all forms of the HPV31 genome (integrated/multimeric, nicked/open circle, episomal, and linear) significantly increased in a time-dependent manner, indicating that the HPV31 genome was amplified in HPV-NOK cells. The data in Fig. 2 demonstrate that our HPV-NOKs express HPV31 transcripts and contain viral episomes that successfully amplify upon differentiation.

### KSHV-NOK and HPV-NOK acquire long spindle-shaped morphology versus uninfected NOK

**Fig 3:**
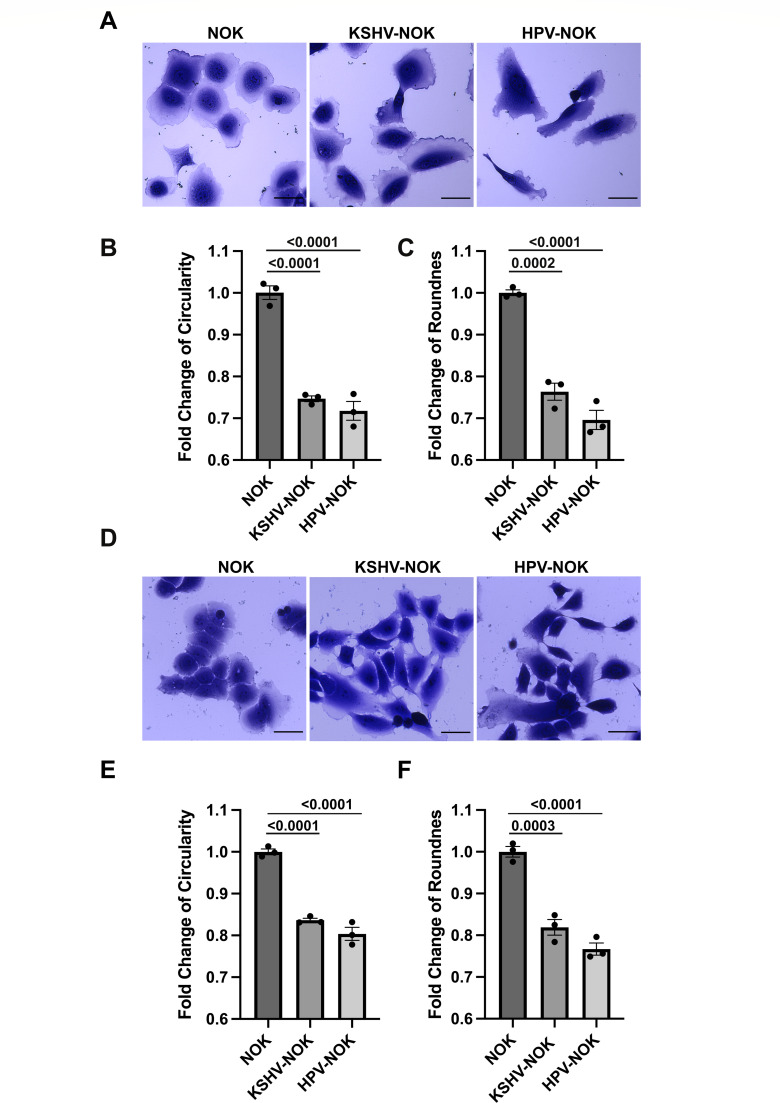
KSHV and HPV31 modulate NOK morphology. NOK, KSHV-NOK, and HPV-NOK cells were seeded on plates and cultured in E media containing 5% FBS and EGF for 24 h. Cells were either (**A through C**) stained with 0.2% crystal violet after 4% PFA fixation or (**D through F**) cultured with serum starvation media (E media without FBS and EGF) for another 4 days prior to crystal violet staining. Images shown were taken using the 20× objective of a Leica DMi8 fluorescent microscope. (**A and D**) Images shown are representative of three independent experiments. Scale bar, 50 μm. Cell circularity (**B and E**) and cell roundness (**C and F**) of approximately 400 to 1200 cells were measured using ImageJ software. Graphs represent the average of three independent experiments, and error bars represent means ± SEM. *P*-values were analyzed using one-way analysis of variance with Dunnett multiple comparisons.

We noticed that KSHV-NOK and HPV31-infected NOK (HPV-NOK) showed different morphology compared to uninfected NOK. We examined the morphology under different conditions such as complete media and under conditions of serum starvation (SS) to determine if the presence or absence of cytokines and growth factors in the media made a difference to the cell morphology. To show cell morphology more clearly, we performed crystal violet staining after paraformaldehyde (PFA) fixation at 24 h post-seeding in E media containing 5% fetal bovine serum (FBS) and epidermal growth factor (EGF) (Fig. 3A through C), as well as 4 days post-SS using E media without FBS and EGF (Fig. 3D through F). As shown in Fig. 3A and D, KSHV-NOK and HPV-NOK both underwent morphologic transition from round clustered epithelial cells to elongated fibroblast-like spindle-shaped cells. Furthermore, the cell-to-cell contact for KSHV-NOK and HPV-NOK was not as tight as that observed in NOK. Quantification of changes in cell circularity and roundness is shown in Fig. 3B and C and E and F, respectively. KSHV-NOK and HPV-NOK both showed significantly lower cell circularity and roundness versus uninfected NOK regardless of the presence or absence of FBS and EGF in culture. We observed similar results when we used commercial serum-free keratinocyte basal media in this experiment (Fig. S1).

### KSHV and HPV31 infection increase cell proliferation of NOKs

**Fig 4:**
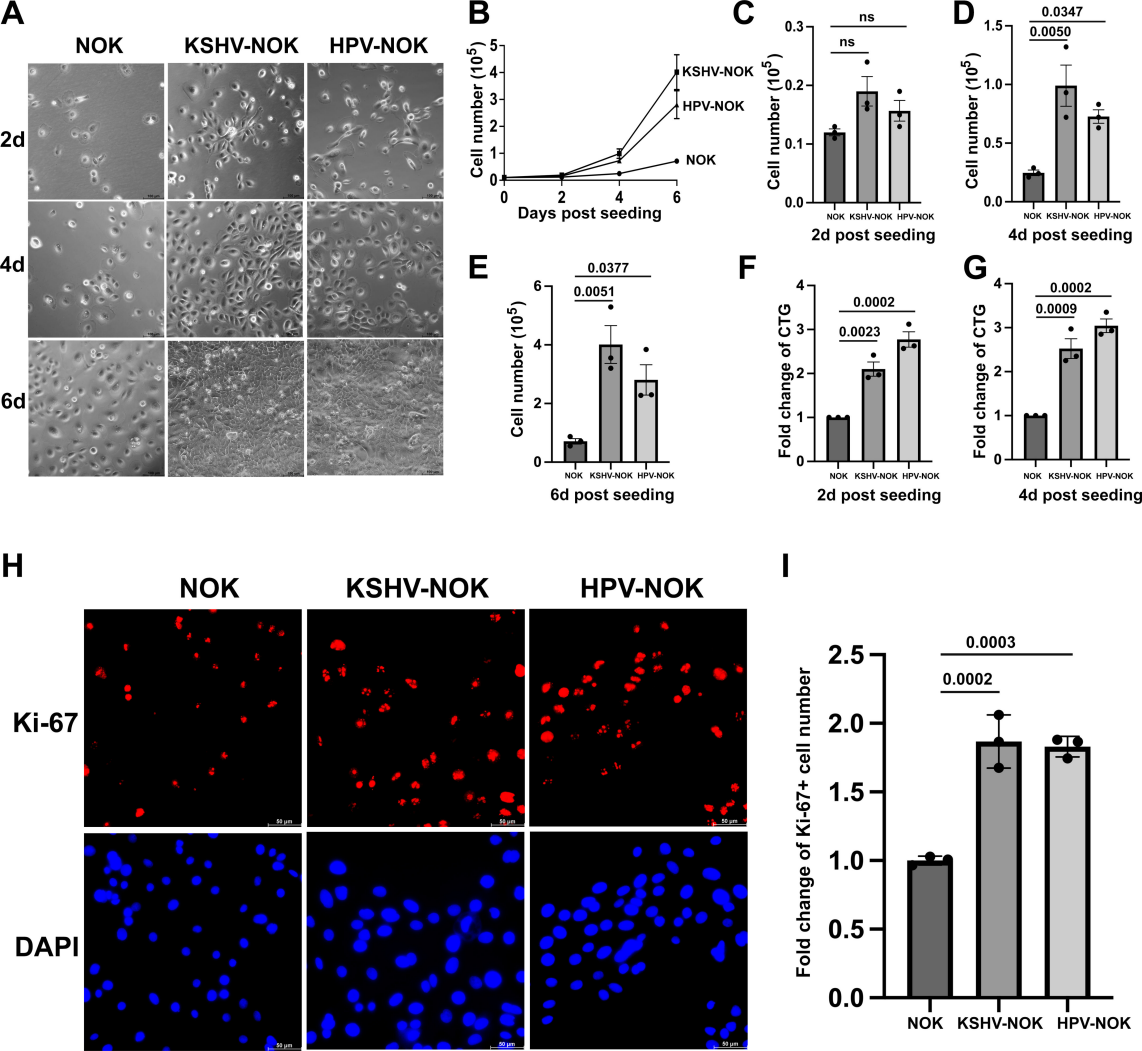
KSHV-NOK and HPV-NOK proliferate faster than NOK in E media. (**A through G**) NOK, KSHV-NOK, and HPV-NOK cells were seeded on plates and cultured in E media containing 5% FBS and EGF for 2 days, 4 days, and 6 days. (**A**) Representative brightfield images of three independent experiments taken under 20× objective of a Leica DMi8 fluorescent microscope are shown, scale bar, 100 μm. (**B through E**) Live cells at different time points were counted after trypan blue staining. Live cell numbers were used to generate growth curves and were analyzed separately at each time point. *P*-values were analyzed using one-way analysis of variance (ANOVA) with Dunnett multiple comparisons. (**F, G**) Cell viability was measured at 2 days and 4 days post-seeding using CTG Luminescent Cell Viability reagent. Graphs represent the average of three independent experiments, and error bars represent mean ± SEM. *P*-values were analyzed using one-way ANOVA with Dunnett multiple comparisons. (**H, I**) NOK, KSHV-NOK, and HPV-NOK cells were seeded onto coverslips in 12-well plates and cultured in E media containing 5% FBS and EGF overnight. Proliferation marker Ki-67 was detected by immunofluorescence assay. Images were taken under 20× and 40× objectives with a Leica DMi8 fluorescent microscopy. Representative 40× images of three independent experiments are shown in panel H, scale bar, 50 μm. Ki-67-positive cells were counted using 20× objective images, and fold change of Ki-67-positive percentage is shown in panel I. Values represent the average of three replicates from three independent experiments, and error bars represent mean ± SEM. *P*-values were analyzed using one-way ANOVA with Dunnett multiple comparisons.

To determine if KSHV or HPV31 infection contributes to oral epithelial cell growth, we seeded equal amounts of NOK, KSHV-NOK, and HPV-NOK into plates to make sub-confluent conditions (10%–15% cell confluence) in E media containing 5% FBS and EGF for 2, 4, and 6 days. At each time point, brightfield images (Fig. 4A) were taken, and the number of viable cells (Fig. 4B through E) was counted after trypan blue staining. As shown in Fig. 4A and B, KSHV-NOK and HPV-NOK proliferated faster than NOK. Based on the growth curves shown in Fig. 4B, the proliferation rates of HPV-NOK and KSHV-NOK were similar. A detailed analysis of viable cell numbers for each time point shown in Fig. 4C through E demonstrates that the increase in growth in KSHV-NOK and HPV-NOK is observed as early as 2 days post-seeding, with an even more significant increase occurring at 4 and 6 days post-seeding. We also measured the cell viability at 2 and 4 days post-seeding using Cell Titer-Glo (CTG) luminescent cell viability reagent. As shown in Fig. 4F and G, a significant increase in cell viability for KSHV-NOK and HPV-NOK also occurred at 2 days post-seeding, and a similar trend for increased viability was held at 4 days post-seeding. In addition, when NOK, KSHV-NOK, and HPV-NOK were grown at sub-confluent conditions in E media containing 5% FBS and EGF, immunofluorescence assays (shown in Fig. 4H and I) confirmed that more KSHV-NOK and HPV-NOK cells expressed Ki-67 (a cellular proliferation marker) compared to uninfected NOK. These results revealed that KSHV and HPV31 infection of NOKs promotes cell proliferation in E media containing FBS and EGF, and the effects were similar between these two viruses.

**Fig 5:**
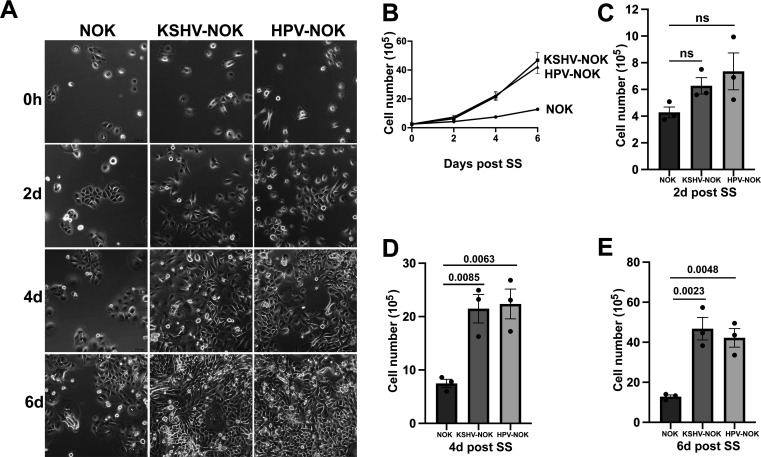
KSHV-NOK and HPV-NOK grow faster than NOK in E media without FBS and EGF. NOK, KSHV-NOK, and HPV-NOK cells were seeded on plates and cultured in E media containing 5% FBS and EGF overnight. Cells were then cultured under conditions of SS media (SS) in E media without FBS and EGF for an additional 2, 4, and 6 days. (**A**) Representative brightfield images of four independent experiments taken with the 20× objective of a Leica DMi8 fluorescent microscope are shown, scale bar, 100 μm. (**B through E**) Live cells were counted at different time points after trypan blue staining. Live cell numbers were used to generate growth curves and were analyzed separately at each time point. Graphs represent the average of three independent experiments, and error bars represent mean ± SEM. *P*-values were analyzed using one-way analysis of variance with Dunnett multiple comparisons.

We next performed a similar cell growth assay under serum starvation conditions by first culturing NOK, KSHV-NOK, and HPV-NOK in E media containing 5% FBS and EGF overnight and then culturing them in serum starvation media using E media without FBS and EGF (Fig. 5) or commercial serum-free keratinocyte basal media (Fig. S2) for another 2, 4, and 6 days. As shown in Fig. 5A and B and Fig. S2A and B, KSHV-NOK and HPV-NOK proliferated faster than NOK in the first 4–6 days post-serum starvation. Figure 5C through E and Fig. S2C through E display the viable cell numbers of HPV-NOK and KSHV-NOK at 2, 4, and 6 days post-seeding compared to uninfected NOK. These results reveal that KSHV and HPV31 infection promotes cell proliferation under conditions of serum starvation.

### KSHV and HPV31 infection enhances NOK cell migration and invasion

**Fig 6:**
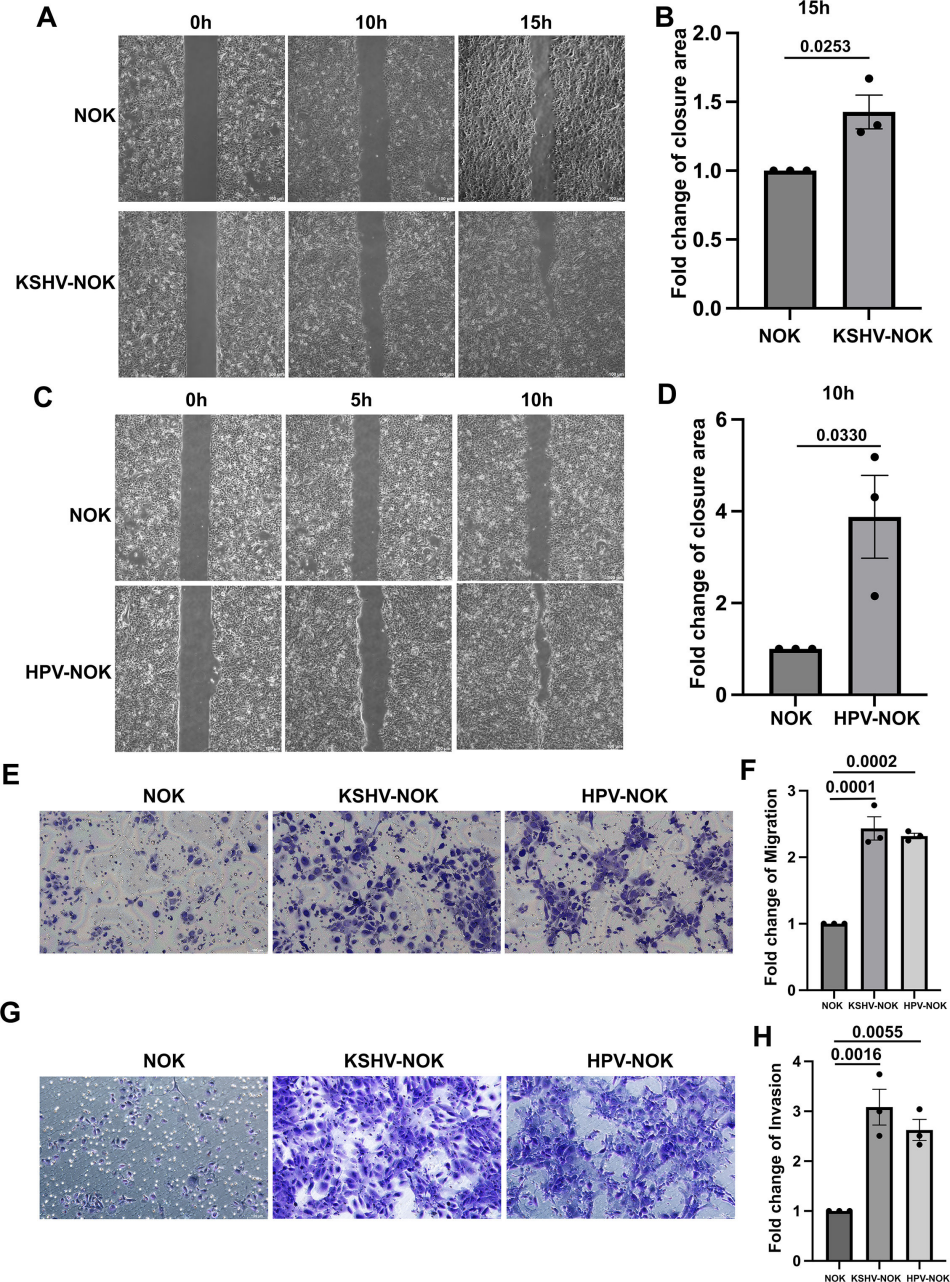
KSHV-NOK and HPV-NOK promote NOK cell migration and invasion. (**A through D**) Wound healing 2D migration assays were performed by seeding NOK and KSHV-NOK cells (**A, B**) or NOK and HPV-NOK cells (**C, D**) into 35 mm two-well culture-insert dishes. After cell attachment, a cell-free gap (wound) was created, after which wound healing (i.e., cell migration) was measured at different time points. Representative brightfield images shown were taken with the 5× objective of a Leica DMi8 fluorescent microscopy, scale bar, 100 µm. Wound closure area was measured using ImageJ software. (**E, F**) A 3D migration assay was performed by seeding NOK, KSHV-NOK, and HPV-NOK on a 24-well plate with 8.0 μm-pore transwell insert without Matrigel. Cells were cultured in serum-free media (E media without FBS and EGF) in the top chamber of the insert, and E media with 5% FBS and EGF was added to the bottom of the well as an attractant. After 16–24 h of migration, cells were fixed with 4% PFA and stained with 0.2% crystal violet. (**G, H**) A 3D invasion assay was performed by seeding NOK, KSHV-NOK, and HPV-NOK into six-well plates with 8.0 μm-pore Matrigel coated-transwell inserts. Cells were cultured in serum-free media (E media without FBS and EGF) in the top chamber of the insert, and E media with 20% FBS and EGF was added to the bottom of the well as attractant. After 30 h of invasion, cells were fixed with 4% PFA and stained with 0.2% crystal violet. Representative images shown in E and G were taken with the 10× objective of a Leica DMi8 fluorescent microscope, scale bar, 100 μm. The number of migrating and invading cells was counted, and fold changes are shown in F and H. Representative images of three independent experiments are shown in panels A, C, E, and G. Graphs represent the average of three independent experiments, and error bars represent mean ± SEM. *P*-value was analyzed using Student’s *t*-test for panels B and D. *P*-value was analyzed using one-way analysis of variance with Dunnett multiple comparisons for panels F and H.

Cell migration and invasion are closely related to tumor metastasis. To further explore the pathogenesis and tumorigenesis in oral epithelial cells by KSHV and HPV31 infection, we examined migration and invasion phenotypes in KSHV-NOK and HPV-NOK compared to uninfected NOK (Fig. 6). Migration was first measured by analysis with a two-dimensional wound healing assay over a 15 h period. As shown in Fig. 6A through D, KSHV-NOK and HPV-NOK significantly enhanced wound closure rates. To rule out the possibility that cell proliferation rates were impacting cell migration, we performed a similar wound healing assay after treatment with mitomycin C (10 µg/mL) for 3 h and obtained similar results (Fig. S3). Second, a three-dimensional migration assay using transwell chambers without Matrigel (Fig. 6E and F) also revealed there were markedly more KSHV-NOKs and HPV-NOKs that had migrated to the bottom membrane of the chambers. A transwell invasion assay using chambers coated with Matrigel was performed next (Fig. 6G and H). The number of invaded KSHV-NOKs and HPV-NOKs was significantly higher compared to uninfected NOK. These findings indicate that KSHV and HPV31 infection facilitate cell migration and invasion of oral epithelial cells.

### KSHV and HPV31 induce EMT in oral epithelial cells

**Fig 7:**
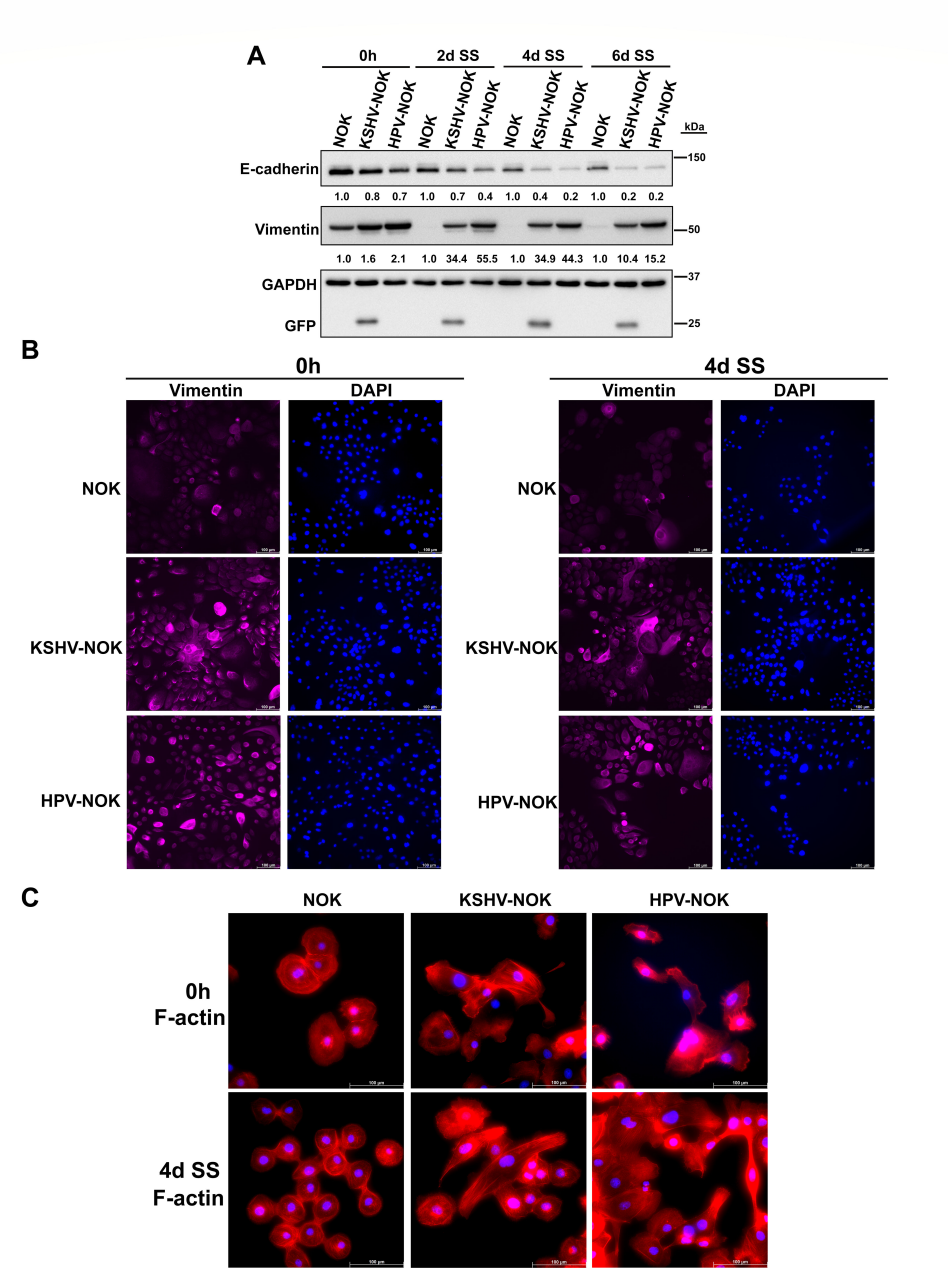
KSHV-NOK and HPV-NOK induce EMT in oral epithelial cells. (**A**) NOK, KSHV-NOK, and HPV-NOK cells were cultured in either E media containing 5% FBS and EGF or serum starvation E media without FBS and EGF for 2, 4, and 6 days. EMT markers were measured by Western blot. (**B, C**) NOK, KSHV-NOK, and HPV-NOK cells were cultured in either E media containing 5% FBS and EGF or serum starvation E media without FBS and EGF for 4 days. Immunofluorescence analysis of vimentin (**B**) and F-actin staining (**C**) was performed. Vimentin images were taken with the 20× objective while F-actin images were taken with the 40× objective of a Leica DMi8 fluorescent microscope. Scale bar for vimentin and F-actin is 100 μm. Panels A, B, and C show representative images of three independent experiments. “SS” indicates serum starvation.

EMT enables epithelial cells to acquire mesenchymal cell phenotypes, such as fibroblast-like cell morphology and cytoarchitecture, increased resistance to apoptosis, and enhanced migratory and invasive capacities (23, 37). To determine whether KSHV and HPV31 infection induces markers of EMT in oral epithelial cells, we screened EMT markers by Western blot (Fig. S4). We found KSHV-NOK and HPV-NOK displayed lower expression levels of the epithelial marker, E-cadherin, but higher levels of expression of the mesenchymal marker, vimentin while ZO-1, beta-catenin, and Slug levels were unchanged. Similar assays were performed to measure levels of E-cadherin and vimentin in both E media containing 5% FBS and EGF and under serum starvation conditions. Both KSHV-NOKs and HPV-NOKs exhibited reduced protein expression of E-cadherin and increased protein expression of vimentin in both E media containing 5% FBS and EGF (0 h) and under serum starvation (2 days, 4 days, and 6 days) culture conditions, using either E media without FBS and EGF (Fig. 7A) or commercial keratinocyte basal media (Fig. S5A). Interestingly, we noted that the levels of E-cadherin tend to gradually decrease in NOK cells at different time points of serum starvation conditions in E media (Fig. 7A) while they appear similar at all time points in the keratinocyte basal media (Fig. S5A). This might suggest that E-cadherin levels in NOK cells may be sensitive to the nutrients or composition of the E media versus the keratinocyte basal media. Like EMT, the ECM is now known to play a key role in many cellular processes including cell proliferation, migration, and differentiation. To determine the effect of KSHV and HPV infection on the ECM, a similar screening assay of ECM markers was performed, and we found that KSHV infection increased MMP9 expression while HPV infection decreased MMP9 expression only in commercial serum-free keratinocyte basal media-treated samples (Fig. S5B). We did not detect any clear MMP9 bands in E media with (Fig. S5B, 0h) or without FBS and EGF (data not shown). To validate EMT results shown above in Western blots assays, we confirmed that KSHV-NOK and HPV-NOK expressed high levels of vimentin by immunostaining assay (Fig. 7B and Fig. S5C). With phalloidin F-actin staining (Fig. 7C and Fig. S5D), we found that KSHV-NOK and HPV31-NOK displayed elongated and spindle-like mesenchymal cell morphology, which was consistent with the findings shown in Fig. 3. These results indicate that KSHV and HPV31 infection induced a partial mesenchymal transition in oral epithelial cells.

### Knockdown of vimentin reduces EMT-associated phenotype in KSHV-NOK and HPV-NOK

**Fig 8:**
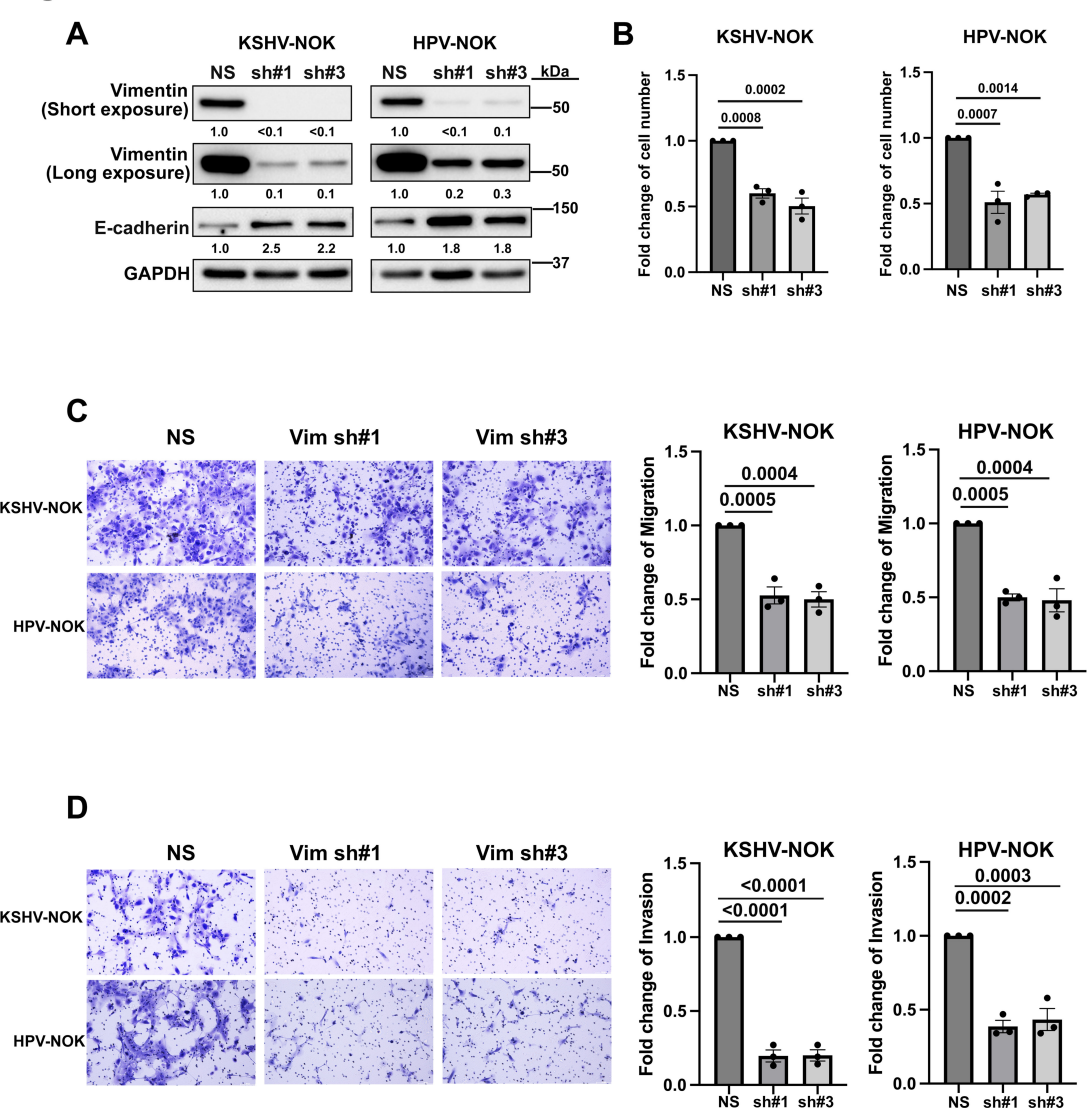
Knockdown of vimentin reduces KSHV-NOK and HPV-NOK cell proliferation, migration, and invasion. (**A**) Expression levels of vimentin and E-cadherin were examined by Western blot in shRNA #1- and #3-transduced KSHV-NOK and HPV-NOK cells, with non-silencing (NS) shRNA performed as a control. (**B**) The effect of vimentin knockdown on KSHV-NOK and HPV-NOK viability was performed using trypan blue staining at 7 days post-transduction. (**C**) The effect of vimentin knockdown on the migration of KSHV-NOK and HPV-NOK cells was detected by a transwell migration assay. (**D**) The effect of vimentin knockdown on invasion of KSHV-NOK and HPV-NOK cells was detected by a transwell invasion assay. Representative images of three independent experiments are shown (A, C left, and D left). Representative images shown in C and D were taken with the 10× objective of a Leica DMi8 fluorescent microscope, scale bar, 100 μm. Graphs (B, C right, and D right) represent the average of three independent experiments, and error bars represent mean ± SEM. *P*-value was analyzed using one-way analysis of variance with Dunnett multiple comparisons.

We found that both KSHV-NOK and HPV-NOK expressed significantly higher levels of vimentin (Fig. 7; Fig. S4 and S5), and vimentin is at the heart of EMT-mediated metastasis (28). To investigate whether vimentin plays a key role in EMT-associated phenotypic regulation by KSHV and HPV31 infection, we performed vimentin knockdown assays with shRNA lentiviruses in KSHV-NOK and HPV-NOK. Vimentin shRNAs resulted in efficient depletion in both KSHV-NOK and HPV-NOK (Fig. 8). Notably, vimentin knockdown led to upregulation of E-cadherin (Fig. 8A). Furthermore, vimentin depletion resulted in significant inhibition of cell proliferation (Fig. 8B), migration (Fig. 8C), and invasion (Fig. 8D) when compared with control shRNA-transduced KSHV-NOK and HPV-NOK. Taken together, these data show that vimentin plays an important role in the regulation of the EMT-associated phenotype by KSHV and HPV31 infection.

### Treatment of eribulin reverses EMT in KSHV-NOK and HPV-NOK

**Fig 9:**
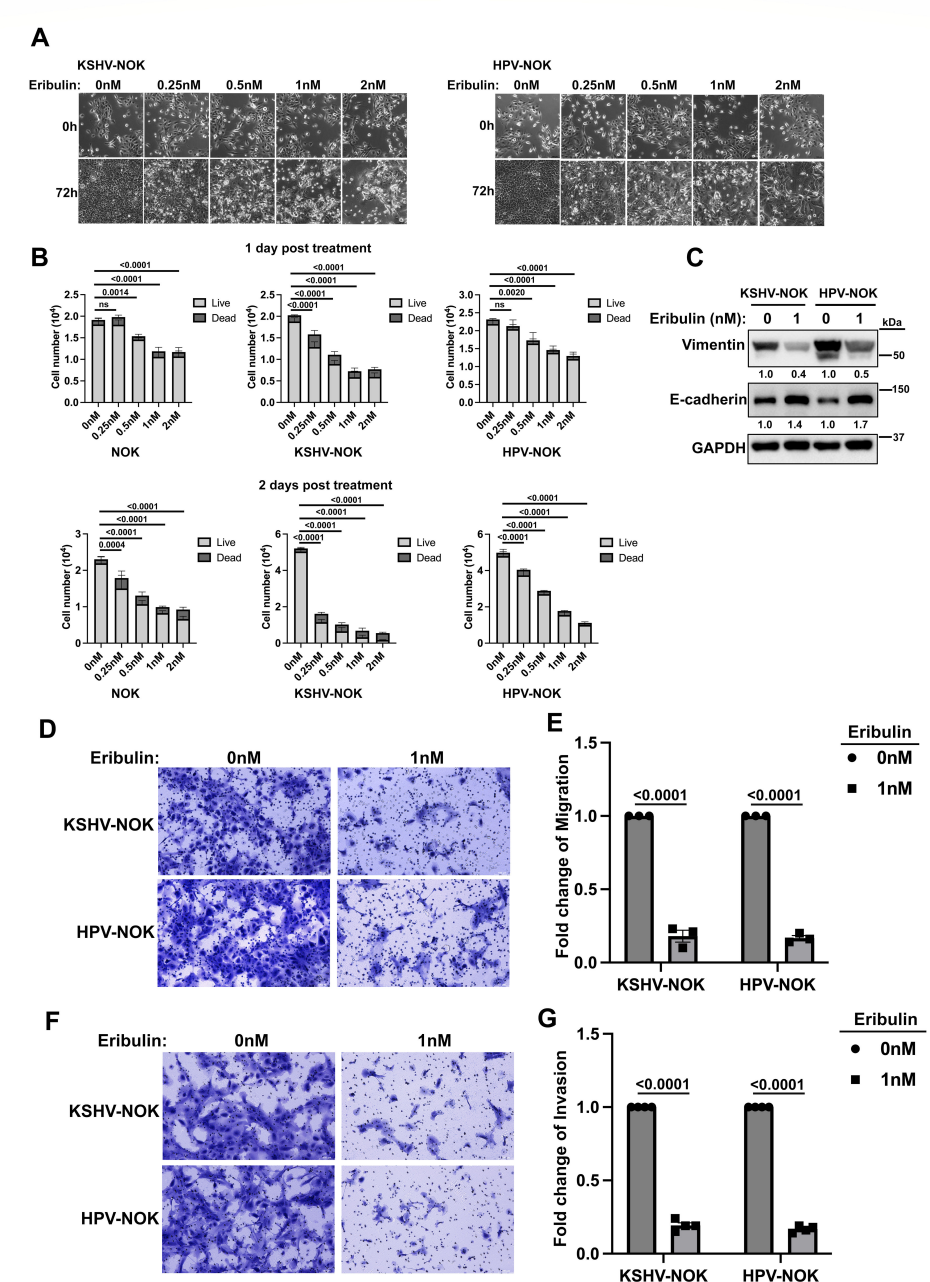
Eribulin treatment reduces KSHV-NOK and HPV-NOK cell proliferation, migration, and invasion. KSHV-NOK and HPV-NOK cells were seeded on plates and cultured in E media containing 5% FBS and EGF overnight. Cells were then cultured in E media with eribulin for the indicated times (**A, B**) or 8 days (**C through G**). (**A**) Representative brightfield images shown were taken with the 20× objective of a Leica DMi8 fluorescent microscope, scale bar, 100 μm. (**B**) Quantification of live and dead cells is depicted. The graph represents the average of triplicate wells, and error bars represent mean ± SD. *P*-values were analyzed using two-way analysis of variance (ANOVA) with Dunnett multiple comparisons. (**C**) EMT markers were detected after KSHV-NOK and HPV-NOK cells were treated with eribulin. (**D**) Migration assay of eribulin-treated-KSHV-NOK and HPV-NOK. Representative images of migrated cells shown were taken under the 10× objective of a Leica DMi8 fluorescent microscope, scale bar, 100 μm. (**E**) Quantification of migrated cells from (**D**) is depicted. (**F**) Invasion assay of eribulin-treated KSHV-NOK and HPV-NOK cells. Representative images of invaded cells shown were taken with the 10× objective of a Leica DMi8 fluorescent microscope, scale bar, 100 μm. (**G**) Quantification of invaded cells from (**F**) is depicted. Panels A, C, and D show representative images of three independent experiments, while panel F shows representative images of four independent experiments. Graph E represents the average of three independent experiments while panel G represents the average of four independent experiments, and error bars represent mean ± SEM. *P*-values were analyzed using two-way ANOVA with Sidak multiple comparisons for panels E and G.

Eribulin is a chemotherapy drug and has previously been reported to reverse EMT and induce mesenchymal-to-epithelial transition (MET) (38). To investigate whether EMT plays an important role in KSHV and HPV31 infection, we carried out EMT inhibition assays with eribulin. We treated KSHV-NOK and HPV-NOK with dimethyl sulfoxide (DMSO) as negative control (0 nM), or 0.25 nM, 0.5 nM, 1 nM, and 2 nM of eribulin for 72 h. Brightfield images are shown in Fig. 9A and demonstrated that eribulin treatment reduced KSHV-NOK and HPV-NOK cell growth. To quantify live and dead cells post-eribulin treatment of the uninfected NOK, HPV-NOK, and KSHV-NOK cells, we performed a similar assay by seeding 1.5 × 10^4^ cells per well in a 24-well plate (Fig. 9B). We treated the cells with different amounts of eribulin for 1 or 2 days and counted live versus dead cells by trypan blue staining. We found that the KSHV-NOK cells were most sensitive to eribulin treatment compared to the uninfected NOK and HPV-NOK cells at all concentrations tested.

Furthermore, as shown in Fig. 9C, eribulin decreased protein levels of vimentin but increased protein levels of E-cadherin, suggesting eribulin reversed EMT and induced MET in KSHV-NOK and HPV-NOK. For migration and invasion assays, we pretreated cells with eribulin for 8 days and then loaded an equal number of cells for assays. We found that eribulin treatment significantly inhibited cell migration and invasion of these cells (Fig. 9D through G). Taken together, our data demonstrate that EMT plays an important role in KSHV and HPV31 infection and that eribulin can reverse EMT and induce MET in KSHV-NOK and HPV-NOK.

### Knockdown of p53 does not impact growth of NOK cells

We sequenced p53 in the uninfected NOK, HPV-NOK, and KSHV-NOK cells and found that uninfected NOK and HPV-NOK have wild-type p53. However, the KSHV-NOK had a single point mutation (R282G) in p53 that may have arisen during cell culture. We next detected p53 expression level by Western blot and found that the KSHV-NOK showed increased p53 protein levels compared to uninfected NOK, while HPV-NOK showed slightly decreased p53 compared to control NOK, likely because HPV E6 targets p53 for degradation (39) (Fig. S6A). To investigate the effect of p53 on the growth of uninfected NOK, HPV-NOK, and KSHV-NOK cells, we transfected two different p53 small interfering RNAs (siRNAs) that target the N terminal of p53 to knock down p53 expression. We confirmed that p53 expression was depleted 3 days post-siRNA transfection, and this did not have any impact on the expression of the EMT marker, vimentin (Fig. S6B). We also found that cell proliferation of p53 siRNA-transfected uninfected NOK, HPV-NOK, and KSHV-NOK was not significantly changed (Fig. S6C). This is likely due to the fact that we are using hTERT-immortalized NOK cells, and constitutive expression of hTERT has previously been reported in the literature to inhibit p53 function. For example, constitutive expression of hTERT can promote anti-apoptotic responses and inactivate the function of the p53 tumor suppressor (40). Additionally, constitutive hTERT expression was also shown to increase cell survival and inhibit wild-type p53-dependent apoptosis in response to mitomycin C or 5-fluorouracil (41). Furthermore, both HPV and KSHV viral proteins have been shown to block p53 function, which may be an additional reason why p53 knockdown did not impact cell proliferation in the virus-infected cells (42, 43).

### Measurement of KSHV and HPV31 lytic genes during serum starvation or eribulin treatment in undifferentiated cells

To investigate if serum starvation or eribulin treatment induces KSHV or HPV31 lytic reactivation in undifferentiated cells, we plated NOK, KSHV-NOK, and HPV-NOK and either serum starved them for 2 days or treated them with eribulin for 2 days. We did not find that either serum starvation or eribulin induced lytic replication of KSHV as measured by RFP-positive KSHV-NOK cells (Fig. S7A) and KSHV lytic protein expression of ORF45 and K8α (Fig. S7B). KSHV-iSLK cells treated with doxycycline and sodium butyrate are shown as a positive control. For HPV31-NOK, E1^E4 and L1 mRNA levels did not change at 2 days post-serum starvation. Following 2 days of eribulin treatment, E1^E4 levels did not change, while L1 mRNA levels increased slightly (Fig. S7C).

## DISCUSSION

The oral cavity is the major route for KSHV infection and transmission (13), and high-risk HPV-associated oropharyngeal cancers have increased in incidence and represent an emerging viral epidemic of cancer (16).

While other researchers have shown KSHV *de novo* lytic infection in oral epithelial cells (44–46), our study is the first to show that KSHV latency in NOK displays sustained KSHV latent gene expression and viral genomes. NOKs also support rKSHV.219 *de novo* lytic infection as demonstrated by almost 100% GFP-positive infected cells and many RFP-positive lytic cells at 24 hpi, 48 hpi, and 72 hpi as previously reported (47). Furthermore, we found that the NOKs support HPV31 early gene expression (e.g., E2, E6, and E7) and maintain HPV31 genomes episomally over long-term culture. Furthermore, we show that NOKs support late viral events as evidenced by HPV31 genome amplification upon high-calcium differentiation (48, 49).

KSHV has previously been shown to induce endothelial–mesenchymal transition (50–52). In terms of epithelial cells, we are the first to show that KSHV latent infection with whole virus can induce EMT in oral epithelial cells. In terms of the impact of HPV on EMT, previous studies have focused on the effect of single oncoproteins, i.e., E6/E7 of HPV16 or HPV18, and their individual impact on EMT, but did not use HPV whole-virus-infected cell lines to examine EMT (53, 54). In contrast, our study is in the context of whole-virus infection and focuses on the impact of HPV31 whole-virus infection of NOK and how the virus modulates EMT properties of the HPV-NOK. We found that both KSHV and HPV31 infection modulate oral epithelial cell characteristics including cell morphology, proliferation, migration, invasion, and EMT. To our knowledge, our study is the first to reveal the impact of these oncoviruses on oral epithelial cells in the context of whole-virus infection.

In terms of EMT, we found that both KSHV-NOK and HPV31-NOK expressed much lower levels of the epithelial cell marker, E-cadherin, and significantly higher levels of the mesenchymal cell marker, vimentin, and reorganized F-actin, indicating that KSHV and HPV31 induce EMT in NOKs. E-cadherin is a well-known tumor suppressor and one of the most important mediators during cell-cell adhesion in epithelial tissues; loss of E-cadherin correlates with EMT induction, increased invasiveness, and metastasis of tumors (55). Vimentin, a cytoskeletal protein, is expressed mainly in cells of mesenchymal origin and involved in multiple cellular mechanical processes including cell migration, adhesion, and division (56–58). EMT (downregulation of E-cadherin combined with upregulation of vimentin) explains the phenotypes observed in our study, wherein the KSHV- and HPV31-infected NOK cells showed fibroblast-like morphology, greater migration, and invasion ability. The KSHV- and HPV31-infected NOK cells also displayed faster proliferation under low cell confluence conditions. Interestingly, KSHV (though not HPV31) latent infection also upregulated MMP9 protein levels in serum-free keratinocyte basal media, while our previous work showed that KSHV K1 protein induced MMP9 expression in endothelial cells (59). Further research is needed to identify whether K1 or other KSHV genes are involved in MMP9 upregulation in our epithelial cell model.

Since vimentin is at the heart of EMT-mediated metastasis (28) and wound healing (60), we investigated the role of vimentin in KSHV- and HPV31-induced EMT in oral epithelial cells. As expected, depletion of vimentin resulted in a significant decrease in proliferation, migration, and invasion of KSHV-NOKs and HPV-NOKs. Notably, depletion of vimentin increased E-cadherin protein levels, indicating that vimentin depletion reversed EMT to a certain degree at the molecular level. Eribulin, an EMT reversal agent, significantly inhibited cell migration and invasion of KSHV- and HPV-infected NOKs. To our knowledge, this is the first study to evaluate the effect of eribulin in the context of HPV- and KSHV-infected cells. In summary, KSHV- and HPV31-induced EMT plays an important role in the proliferation, migration, and invasion of oral epithelial cells and suggests that EMT may serve as a potential therapeutic target for KSHV- and HPV-associated oral cancer.

In this study, we used hTERT-immortalized NOKs because of their long-term survival advantage over primary NOK cells. It is important to note that the EMT phenotypes of the KSHV-NOK and HPV-NOK were obtained using these hTERT-immortalized NOK cells. Hence, a future goal is to examine these EMT properties in primary NOK cells.

Previous studies have reported that KSHV can infect primary keratinocytes and that the virus establishes a latent infection in primary keratinocytes. However, none of these publications examined EMT or investigated EMT markers in the context of whole KSHV virus infection of primary keratinocytes (44, 47, 61). Some studies overexpressing single KSHV proteins have shown that KSHV LANA can modulate EMT and that KSHV LANA increases the migration and invasion potential of the MDA-MB-231 human breast cancer cell line (32). Similarly, for HPV31, to our knowledge, there is no study that has reported that an HPV31-infected hTERT-immortalized-NOK or an HPV31-infected primary oral epithelial cell line can induce EMT. Some studies have shown that E6/E7-immortalized primary epithelial cell lines, including primary human foreskin keratinocytes or primary gingival keratinocytes that express single oncoproteins like HPV E6 or HPV E7 (but not the whole HPV virus), can induce EMT (53, 62).

## MATERIALS AND METHODS

### Cell culture and generation of KSHV latently infected NOK and HPV31-positive stable NOK cell line

NOKs are hTERT-immortalized oral epithelial cells, kindly provided by Dr. Karl Munger (22). 3T3 J2 fibroblast feeder cells, HFKs stably maintaining HPV31 genomes (HPV-HFK) and 293TT cells were previously described(36, 63). For KSHV-NOK generation, NOKs were *de novo* infected with purified recombinant KSHV (rKSHV.219) and then selected by puromycin for at least 3 weeks before KSHV genome copies were measured, and LANA staining was performed. The KSHV-NOK was always cultured with puromycin in the media. For the generation of the HPV31-positive NOK cell line (HPV-NOK), NOKs were co-transfected with HPV31 genome (pBR322-HPV31) and the selection marker (pSV2-Neo). Cells were then cultured with G418 selection for at least 3 weeks. After G418 selection of the HPV-NOK for at least 3 weeks, HPV31 episomes were validated, after which the HPV-NOK cells were routinely cultured without G418 selection. HFKs stably maintaining HPV31 episomes (HPV-HFK) were generated as described previously (64). All epithelial cell lines, NOK, KSHV-NOK, HPV-NOK, and HPV-HFK, were routinely cultured in E media supplemented with 5% FBS (Sigma, F2442) and 5 ng/mL mouse EGF (Corning, 354010) and co-cultured with mitomycin C-treated 3T3 J2 fibroblast feeder cells, as previously described (65). J2 cells were removed from these epithelial cell lines by treatment with Versene buffer (0.5 mM EDTA in Dulbecco’s phosphate buffered saline (DPBS) , pH 8.0) before seeding NOKs into plates or dishes for assays. Unless otherwise stated, all experiments throughout our study were performed using NOKs seeded in the plates or dishes without J2 fibroblast co-culture. J2 cells were grown in Dulbecco’s modified Eagle medium (DMEM) (without sodium pyruvate) (Gibco, 11965-092) supplemented with 10% heat-inactivated bovine serum (Gibco, 26170-035) and 1% Pen-Strep. 293TT and KSHV-iSLK cells were maintained in DMEM (Corning, 10-013-CV) supplemented with 10% FBS (Sigma, F2442) and 1% Pen-Strep. All cell lines were regularly grown in a 37°C and 5% CO_2_ incubator and checked for mycoplasma contamination.

### Western blot

Whole-cell lysates were prepared in cell lysis buffer (50 mM Tris-HCl [pH 7.5], 150 mM NaCl, 5 mM EDTA, 1% Triton X-100, 0.1% SDS) containing protease and phosphatase inhibitor cocktail (Thermo Scientific, 1861281). The protein concentration was measured using a BCA protein assay kit (Thermo Scientific, 23227) according to the manufacturer’s protocol. Whole-cell lysates were next resuspended in 6× SDS-PAGE loading buffer (0.5 M Tris-HCl [pH 6.8], 12% SDS, 0.6 M dithiothreitol (DTT) , 60% glycerol, 0.06% bromophenol blue) and boiled for 10 min. Equivalent amounts of protein were separated by SDS-PAGE and subsequently transferred to 0.45 µm nitrocellulose membranes (Bio-Rad,1620115). Membranes were blocked in 5% nonfat milk in Tris buffered saline Tween (TBST) (50 mM Tris-HCl [pH 7.5], 150 mM NaCl, 0.1% Tween 20) for at least 1 h at room temperature, followed by incubation overnight at 4°C with primary antibody. Primary antibodies used in Western blots were involucrin (Sigma, 19018), TGM1 (Proteintech, 12912-3-AP), K10 (Proteintech, 18343-1-AP),glyceraldehyde phosphate dehydrogenase (GAPDH) (OriGene, TA890003S), E-cadherin (Invitrogen, 33-4000), vimentin (Proteintech, 10366-1-AP), ZO-1 (Cell Signaling Technology, 8193T), beta-catenin (Cell Signaling Technology, 8480T), Slug (Cell Signaling Technology, 9585T), GFP (Abcam, ab290-50), MMP9 (Cell Signaling Technology, 13667S), and p53 (Invitrogen, MA5-12557). Membranes were washed three times (5 min each) in TBST and incubated in secondary antibody for 1 h at room temperature. Secondary antibodies used in Western blots were horseradish peroxidase (HPR)-conjugated anti-rabbit IgG (Cell Signaling Technology, 7074S) and HPR-conjugated anti-mouse IgG (Cell Signaling Technology, 7076S). Membranes were washed three times in TBST. Protein bands were visualized in a ChemiDoc Touch Imaging System (Bio-Rad) using either SuperSignal West Pico PLUS Chemiluminescent Substrate (Thermo Scientific, 34580) or ProtoGlow ECL reagent (National Diagnostics, CL-300). Western band intensity was quantified using ImageJ software.

### RT-qPCR and KSHV intracellular viral genome quantification

For target gene mRNA level quantification, total RNA was isolated from cells using the RNeasy Plus Mini Kit (Qiagen, 74136) according to the manufacturer’s protocol. Reverse transcription of DNase I-treated (DNA-free) RNA was carried out using the SensiFAST cDNA Synthesis Kit (Bioline, 65054) to produce cDNA, which was subjected to qPCR in SensiFast SYBR Lo-ROX Kit (Bioline, 94020) on a QuantStudio 6 Flex real-time PCR machine. Expression of target gene was calculated from the threshold cycle values and normalized to the housekeeping gene GAPDH. Data shown are average fold changes (2^-ΔΔCt^). For KSHV intracellular viral genome quantification, NOK and KSHV-NOK cells were pelleted. Total DNA was isolated using the DNeasy Blood and Tissue kit (Qiagen; 69506) and subjected to qPCR as described above with KSHV ORF39 and GAPDH qPCR primers. Serial dilutions of pCDNA4/TO-*ORF39*-2XStrep (a kind gift from Dr. Britt Glaunsinger, Addgene, 136200) (66) were used to create standard curves. All qPCR primers are listed in Table 1.

**TABLE 1:** RT-qPCR and vimentin shRNAs primers sequences^*a*^

Primer	Sequence（5′−3′）
KSHV-LANA-F	CGGAGCTAAAGAGTCTGGTG
KSHV-LANA-R	GCAGTCTCCAGAGTCTTCTC
KSHV-vFlip-F	TGCGACCTGCACGAAACA
KSHV-vFlip-R	TTACGAGGTTCTCTGTGAGG
KSHV-vCyclin-F	CATCGCATCCCAATATGCTTGC
KSHV-vCyclin-R	CATTGCCCGCCTCTATTATCA
KSHV-Kaposin-F	AACAGACAAACGAGTGGTGGTATC
KSHV-Kaposin-R	GTTTGTGGCAGTTCATGTCC
KSHV-ORF39-F	GGTTTCCCCTGCTACTTCAA
KSHV-ORF39-R	CATGCTTGGCCCGATATAC
HPV31-E2-F	CGGGTGGTCAGGTAATTGTTT
HPV31-E2-R	GGTTTTGGAATTCGATGTGGT
HPV31-E6-F	CCTGCAGAAAGACCTCGGAA
HPV31-E6-R	TGGTGTGTCGTCCCTATATACTATTG
HPV31-E7-F	ACACCTACGTTGCAAGACTATG
HPV31-E7-R	CGAATATCTACTTGTGTGCTCTGT
HPV31-E1^E4-F	TGTTAATGGGCTCATTTGGAA
HPV31-E1^E4-R	GGTTTTGGAATTCGATGTGG
HPV31-L1-F	GCAAACCACCTATTGGAGAGC
HPV31-L1-R	GCTCCAAAGCCTGTATCAACC
GAPDH-F	CGTCATGGGTGTGAACCATGAGAA
GAPDH-R	GAGTCCTTCCACGATACCAAAGTT
Vimentin shRNA #1-F	GATCC**GCTAACTACCAAGACACTATT**CTCGAG**AATAGTGTCTTGGTAG**TTAGCTTTTTA
Vimentin shRNA #1-R	CGCGTAAAAA**GCTAACTACCAAGACACTATT**CTCGAG**AATAGTGTCTTGGTAGTTAGC**G
Vimentin shRNA #3-F	GATCCGCA**GGATGAGATTCAGAATATCTCGAGATAT**TCTG**AATCTCATCCTGC**TTTTTA
Vimentin shRNA #3-R	CGCGTAAAAA**GCAGGATGAGATTCAGAATAT**CTCGAG**ATATTCTGAATCTCATCCTGC**G

^*a*^ Vimentin shRNA target sequences are indicated in bold.

### Calcium-induced differentiation

Calcium-induced differentiation assays were performed as previously described (64). A total of 10 × 10^6^ HPV-NOKs were seeded into 10 cm dishes and co-cultured with 1 × 10^6^ mitomycin C-treated J2 cells in E media containing 5% FBS and EGF. After 24 h, cell confluency was confirmed to be sub-confluent (approximately 80%), and undifferentiated cells (0 h sample) were harvested after removing J2 using Versene buffer while the remaining plates were serum starved for 24 h by replacing media using serum-free keratinocyte basal media (Lonza, CC-3101) containing supplements (Lonza, CC-4131) but without FBS. Cells were then differentiated for 48 h (48 h sample) and 96 h (96 h sample) in high-calcium media (serum-free keratinocyte basal media containing 1.8 mM CaCl_2_, but without any supplements or FBS), as described previously (64). At 48 h post-differentiation induction, the 48 h sample was harvested, and media was changed in the 96 h plate with high-calcium media. At the indicated differentiation time points, cells were collected in the same way as for the 0 h sample. Cell pellets for each time point were divided into two aliquots (one for Southern blot and another for Western blot).

### Southern blot

Total genomic DNA isolation and Southern blot analysis were conducted as described (67). Briefly, epithelial cells were collected after removing J2 feeder cells and lysed in 3 mL of DNA lysis buffer (400 mM NaCl, 10 mM Tris-HCl [pH 7.4], 10 mM EDTA), then 5 µL of RNase A (Sigma, R4642-50MG, 20-40 mg/mL), 30 µL of 20% SDS, and 15 µL of 10 mg/mL proteinase K were added, followed by incubation at 37°C overnight. Genomic DNA was sheared by passing it through an 18-gauge needle approximately 10 times and extracted with phenol-chloroform, followed by ethanol precipitation. Five micrograms of genomic DNA was digested with *Dpn*I (to remove any residual parental HPV31 genome DNA) and *Bam*HI (does not cut the HPV31 genome) or *Dpn*I and *Eco*RV (cuts the HPV31 genome once). Digested DNA was resolved on a 0.8% agarose gel for approximately 16 h at 35V and transferred to a nylon membrane (Immobilon-Ny+; EMD Millipore, INYC00010). The DNA was fixed to the membrane with UV irradiation and then hybridized to a ^32^P-labeled linearized HPV31 genome DNA probe. The membrane was visualized by autoradiography.

### Cell morphology staining

Cells were seeded on plates and cultured in E media containing 5% FBS and EGF for 24 h, or under serum starvation conditions for an additional 4 days in either E media without FBS and EGF or commercial serum-free keratinocyte basal media (Lonza, CC-3101). At harvest time points, media was removed, and cells were washed twice with DPBS. Cells were fixed in 4% methanol-free PFA (Electron Microscopy Sciences, 15710) for 15 min followed by two washes with DPBS. Cells were stained with 0.2% crystal violet for 25 min and washed three times with distilled deionized water. Random-field pictures of each well were imaged with a Leica DMi8 fluorescent microscope using a 20× objective lens. Cell circularity and roundness were measured using ImageJ software.

### Cell growth and viability assay

For the cell growth assay, 1 × 10^4^ cells were seeded on 24-well plates. At the indicated cell growth measurement time points, cell images were taken via Leica DMi8 fluorescent microscopy and collected after trypsin treatment. Live cells were counted manually with a hemocytometer after trypan blue staining. For the cell viability assay, 200 µL (0.5 × 10^4^) cells were seeded on 48-well plates. At indicated cell viability measurement time points, cellular metabolic activity was detected using CTG luminescent cell viability reagent (Promega, G7573) based on the manufacturer’s protocol. Briefly, the plate was equilibrated to room temperature for 30 min prior to the addition of 50 µL of CellTiter-Glo buffer-substrate mixture. Contents were mixed for 2 min by putting the plate on an orbital shaker to induce cell lysis. The plate was allowed to incubate for 10 min at room temperature to stabilize the luminescent signal. All contents were transferred from the 48-well plate to a 96-well solid white plate (Greiner, 655083). Absorbance was measured on a CLARIOstar Plus plate reader (BMG Labtech).

### Immunofluorescence assay

Cell media was removed, and cells were washed twice with DPBS followed by fixation in 4% methanol-free PFA for 15 min. Cells were permeabilized in 0.25% Triton X-100 for 10 min and then blocked with 3% bovine serum albumin for 2 h at 37°C prior to primary antibody incubation overnight at 4°C. Primary antibodies used in immunofluorescence assays were LANA (Advanced Biotechnologies, 13-210-100), Ki-67 (Cell Signaling Technology, 9449S), and vimentin (Proteintech, 10366-1-AP). Cells were washed three times (5 min each) in DPBS and incubated in secondary antibody for 1 h at room temperature. Secondary antibodies used in immunofluorescence assays were Cy3 goat anti-rat IgG (Invitrogen, A10522) for LANA, Alexa Fluor 568 goat anti-mouse IgG (Invitrogen, A11004) for Ki-67, and Alexa Fluor 647 goat anti-rabbit IgG (Invitrogen, A21244) for vimentin. Cells were washed three times in DPBS (10 min each) and then stained by DAPI (Sigma, MBD0015) for 15 min followed by three washes with DPBS. For plates with coverslips, coverslips were then mounted onto slides using ProLong Diamond Antifade Mountant (Invitrogen, P36961). The edges of coverslips were sealed using nail polish. Samples were imaged on the Zeiss LSM 700 confocal microscope or Leica DMi8 fluorescent microscope.

### Vimentin shRNA lentivirus generation and transduction

Vimentin shRNA and lentivirus generation and transduction were performed based on a previous report (68). Briefly, vimentin shRNA primers were annealed and cloned into the lentivector pYNC352 (kind gift from Dr. John Nicholas) using *Bam*HI and *Mlu*I restriction sites. Vimentin shRNA #1 and #3 sequences are listed in Table 1.

For lentivirus generation, 8 × 10^6^ 293TT cells were plated into 10 cm dishes of DMEM containing 10% FBS (Sigma, F2442) without any antibiotics. At 16–18 h post-seeding, cell confluence was approximately 70%–80%, and cells were co-transfected with 8 µg pYNC352-shRNA plasmid NS (non-silencing control) or pYNC352-vimentin shRNAs, 6 µg psPAX2 (Addgene, 12260, kind gift from Dr. John Nicholas), and 2 µg pMD2.G (Addgene, 12259, kind gift from Dr. John Nicholas) using FuGENE HD Transfection Reagent (Promega, E231A) according to the manufacturer’s protocol. At 6–8 h post-transfection, media was changed to DMEM containing 10% FBS (Sigma, F2442) and 1% Pen-Strep, and cells were incubated for an additional 48 h. Lentivirus-containing media was collected and concentrated by ultracentrifugation in an SW-32 Ti rotor (Beckman Coulter) at 25,000 rpm for 2.5 h at 4°C. The supernatant was discarded, and 3 mL–5 mL of basal E media without FBS or EGF was added to the viral pellets and left at 4°C overnight. Aliquots of resuspended virus were stored at –80°C.

For lentivirus transduction, cells were plated 16–18 h prior to transduction. Cells were transduced with concentrated lentivirus in the presence of 5 µg/mL polybrene for 16–18 h, then media containing virus and polybrene was removed and replaced with fresh media. Since the lentivector pYNC352 can express GFP, equivalent transduction efficiency between NS and vimentin shRNAs was confirmed by percentage of GFP-positive cells at 2 days post-transduction.

### Wound healing assay

Cells were suspended in complete culture media at 5 × 10^5^ cells/mL, and 70 µL cell suspension was seeded into each chamber of the culture insert (Ibidi, 81176). After the appropriate duration for cell attachment (16–18 h), cells were treated with or without 10 µg/mL of mitomycin C for 3 h. A wound (cell-free gap) was created by removal of the insert using sterile tweezers followed by two washes with DPBS. The complete media was added, and images were taken at 5× magnification using a Leica DMi8 fluorescent microscope every 5 h after insert removal. The wound area (cell-free space) in random fields was measured using ImageJ software.

### Transwell migration assay

Cells were collected and resuspended in E media without FBS or EGF at a density of 2 × 10^5^/mL, and 200 µL cell suspension (4 × 10^4^ cells) was loaded into the transwell insert with 8.0 µm pores in a 24-well plate (Corning, 3464). A total of 750 µL complete E media containing 5% FBS and EGF was added to the lower chamber. After about 24 h, cells were washed with DPBS twice and fixed in 4% methanol-free PFA for 15 min. The insert was stained with 0.2% crystal violet for 25 min and washed three times with distilled, deionized water. Cells that did not migrate were removed from the interior of the insert with a sterile cotton swab. Random areas of each insert were imaged on a Leica DMi8 fluorescent microscope with the 10× objective, and migrated cells were counted using Photoshop software.

### Transwell invasion assay

Matrigel-coated transwell inserts (Corning, 354481) were rehydrated for 2 h using DMEM media in a cell incubator. Cells were collected and resuspended in E media without FBS or EGF at a density of 2.5 × 10^5^/mL, and 2 mL cell suspension (5 × 10^5^ cells) was loaded into the transwell insert. Complete E media containing 20% FBS and EGF was added to the lower chamber. After about 24–30 h, invaded cells were stained and counted similarly as described above in the transwell migration assay.

### Rhodamine phalloidin F-actin staining

A 200× stock solution of rhodamine phalloidin (Invitrogen, R415) was prepared by dissolving the vial contents in 300 µL methanol. Cells on coverslips were washed twice with pre-warmed DPBS and fixed with 4% methanol-free PFA for 15 min. Cells were washed two or three times with DPBS and permeabilized in 0.25% Triton X-100 for 10 min, followed by two or three washes with DPBS. F-actin was stained with 1:200 diluted rhodamine phalloidin stock solution for 30 min at room temperature. Cells were then stained with DAPI for 15 min followed by three washes with DPBS. Coverslips were mounted onto slides using ProLong Diamond Antifade Mountant (Invitrogen, P36961). The edges of coverslips were sealed using nail polish. Samples were imaged on a Leica DMi8 fluorescent microscope at 40× objective.

### Eribulin treatment and phenotypic assays

Eribulin mesylate was purchased from Med Chem Express (catalog number is HY-13442A). Eribulin was dissolved in DMSO for a 5 mM stock solution. Aliquots were stored in −80°C. Eribulin treatment and phenotypic assays were performed based on a previous report (38). For cell growth assays by trypan blue staining, equivalent numbers of cells were seeded in triplicate (10%–30% confluency) and then were treated in E media with DMSO or different doses of eribulin. Live and dead cell numbers were manually counted using trypan blue staining at the indicated time points post-treatment.

For Western blot, migration, and invasion assays, KSHV-NOK and HPV-NOK were seeded and cultured overnight. Cells (~50% cell confluency) were treated with E media containing eribulin for 8 days while E media with DMSO was used as vehicle control. Cells were split and consistently cultured in E media with DMSO or eribulin. At 8 days post-eribulin treatment, cells were collected for Western blot, or equivalent cells were re-seeded into plate for migration and invasion assays based as described above.

### p53 sequencing and siRNA knockdown assays

To evaluate the TP53 protein status (p53 sequencing), three data sets—NOK-HPV, NOK-KSHV, and NOK—were analyzed using long-read sequencing data. Genomic DNA was extracted from 1 million cultured cells per condition. High-fidelity (HiFi) long-read sequencing was performed using PacBio technology. The TP53 gene was defined according to the GRCh38.p14 Primary Assembly, located on the complement strand of Homo sapiens chromosome 17, coordinates NC_000017.11:7,668,421–7,687,490. FASTQ-formatted PacBio HiFi reads were aligned to the TP53 reference sequence (refSeq.fasta) using Minimap2 (macOS build), optimized for genome assembly. The alignment was performed using the following parameters: -x asm10 (assembly mode for accurate long reads), --frag=yes (to detect fragment alignments), --secondary=no (to exclude secondary alignments), and -k21 (21-mer for indexing). Output was generated in SAM format. NOK-HPV: 3,058,533 total reads; 31 reads aligned to TP53. NOK-KSHV: 2,996,010 total reads; 26 reads aligned to TP53. NOK (control): 2,850,142 total reads; 17 reads aligned to TP53.

For p53 knockdown assays, two ON-TARGETplus Human TP53 individual siRNAs (#1 is Dharmacon, J-003329-15-0010: GUGCAGCUGUGGGUUGAUU, and #2 is Dharmacon, J-003329-16-0010: GCAGUCAGAUCCUAGCGUC) were used to deplete p53 in NOK, KSHV-NOK, and HPV-NOK. The ON-TARGETplus Non-targeting Control siRNA #1 (Dharmacon, D-001810-01-50: UGGUUUACAUGUCGACUAA) was used as the non-targeting control (NS). The siRNAs were reconstituted in 1× siRNA buffer (diluted from 5× siRNA buffer [Dharmacon, B-002000-UB-100] in water) for a 100 µM stock solution. Aliquots of 20 µL siRNAs were stored in −20°C. NOK, KSHV-NOK, and HPV-NOK cells were seeded at a density of 5 × 10^4^ cells per well in a 12-well plate in E media containing 5% FBS and EGF. Sixteen to 18 h later, cells (~20% cell confluency) were transfected with 2 µL siRNAs per well using Lipofectamine RNAiMAX Reagent (Invitrogen, 13778150). Media was changed at ~10 h post-transfection. Transfected cells were cultured for 3 days and then harvested for Western blot to confirm p53 knockdown efficiency or for live cell number counting by trypan blue staining.

### Statistical analysis

All data were generated and analyzed with GraphPad Prism 10. Student’s *t*-test was used to compare the mean between two groups. Analysis of variance followed by a *post hoc* corrected for multiple comparisons was used to compare three or more groups. The chosen *post hoc* multiple comparisons tests were performed as recommended by GraphPad Prism 10. Specific tests for each data set are indicated in the figure legends. *P*-values <0.05 were considered significant. “ns” indicates non-significant *P*-values ≥ 0.05.

## SUPPLEMENTAL MATERIAL

Figure S1 mbio.00484-25-s0001.tif KSHV and HPV31 modulate NOK morphology in serum-free keratinocyte basal medium.

Figure S2 mbio.00484-25-s0002.tif KSHV-NOK and HPV-NOK grow faster than NOK in serum-free keratinocyte basal medium.

Figure S3 mbio.00484-25-s0003.tif KSHV-NOK and HPV-NOK promote wound healing.

Figure S4 mbio.00484-25-s0004.tif Screening of EMT markers in NOK, KSHV-NOK, and HPV-NOK.

Figure S5 mbio.00484-25-s0005.tif KSHV-NOK and HPV-NOK induce EMT in serum-free keratinocyte basal medium culture environment.

Figure S6 mbio.00484-25-s0006.tif Effect of p53 on NOK, KSHV-NOK, and HPV-NOK cell growth.

Figure S7 mbio.00484-25-s0007.tif Measurement of KSHV-NOK and HPV-NOK lytic gene expression.

Legends mbio.00484-25-s0008.docx Supplemental figure legends.

ASM does not own the copyrights to Supplemental Material that may be linked to, or accessed through, an article. The authors have granted ASM a non-exclusive, world-wide license to publish the Supplemental Material files. Please contact the corresponding author directly for reuse.
