# Supplementary figures and images for: KSHV and HPV modulate epithelial-to-mesenchymal transition in oral epithelial cells
Source: mBio. 2025 Aug 15;16(9):e00484-25. doi: 10.1128/mbio.00484-25 (PMC12421897; doi:10.1128/mbio.00484-25)

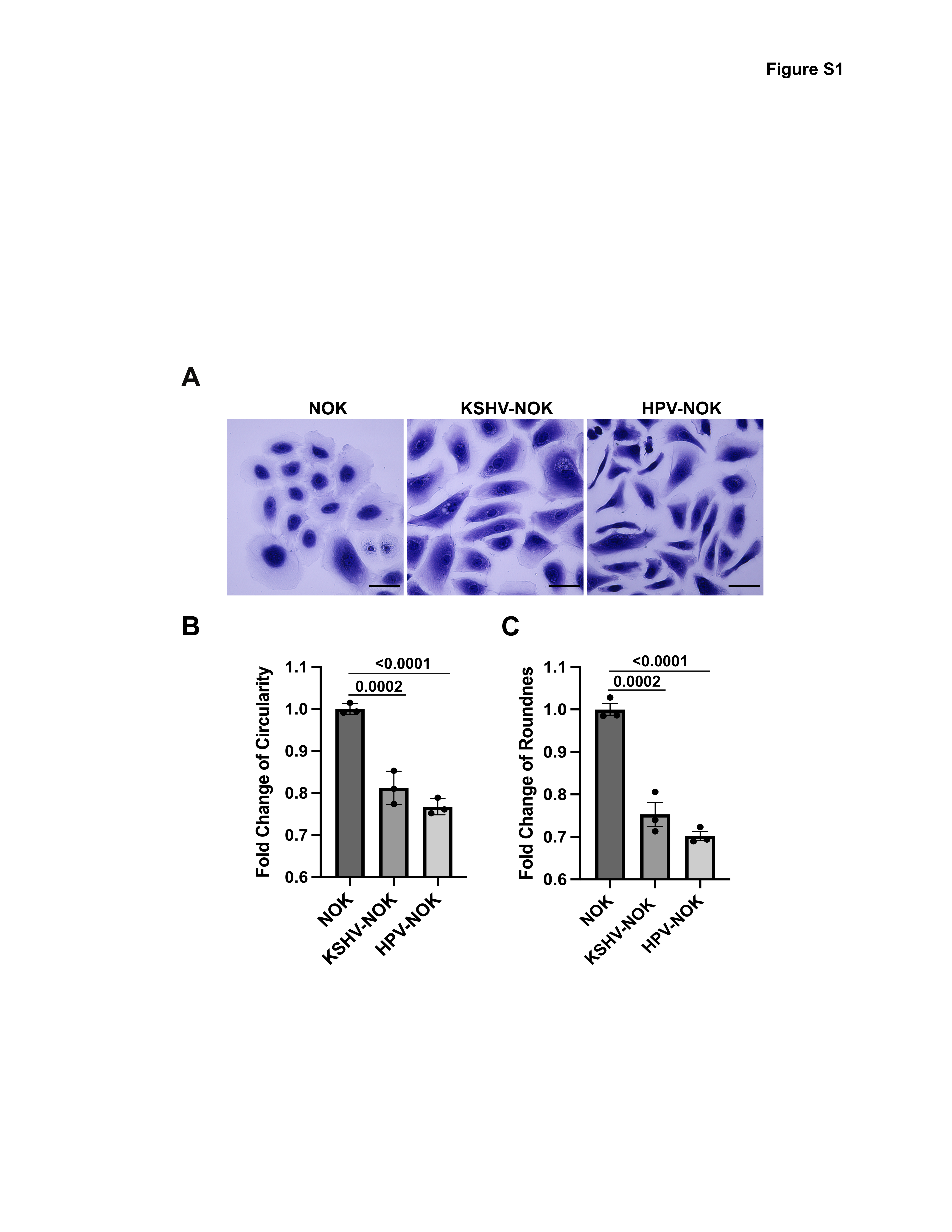

Supplement: Figure S1 — KSHV and HPV31 modulate NOK morphology in serum-free keratinocyte basal medium. [file mbio.00484-25-s0001.tif]

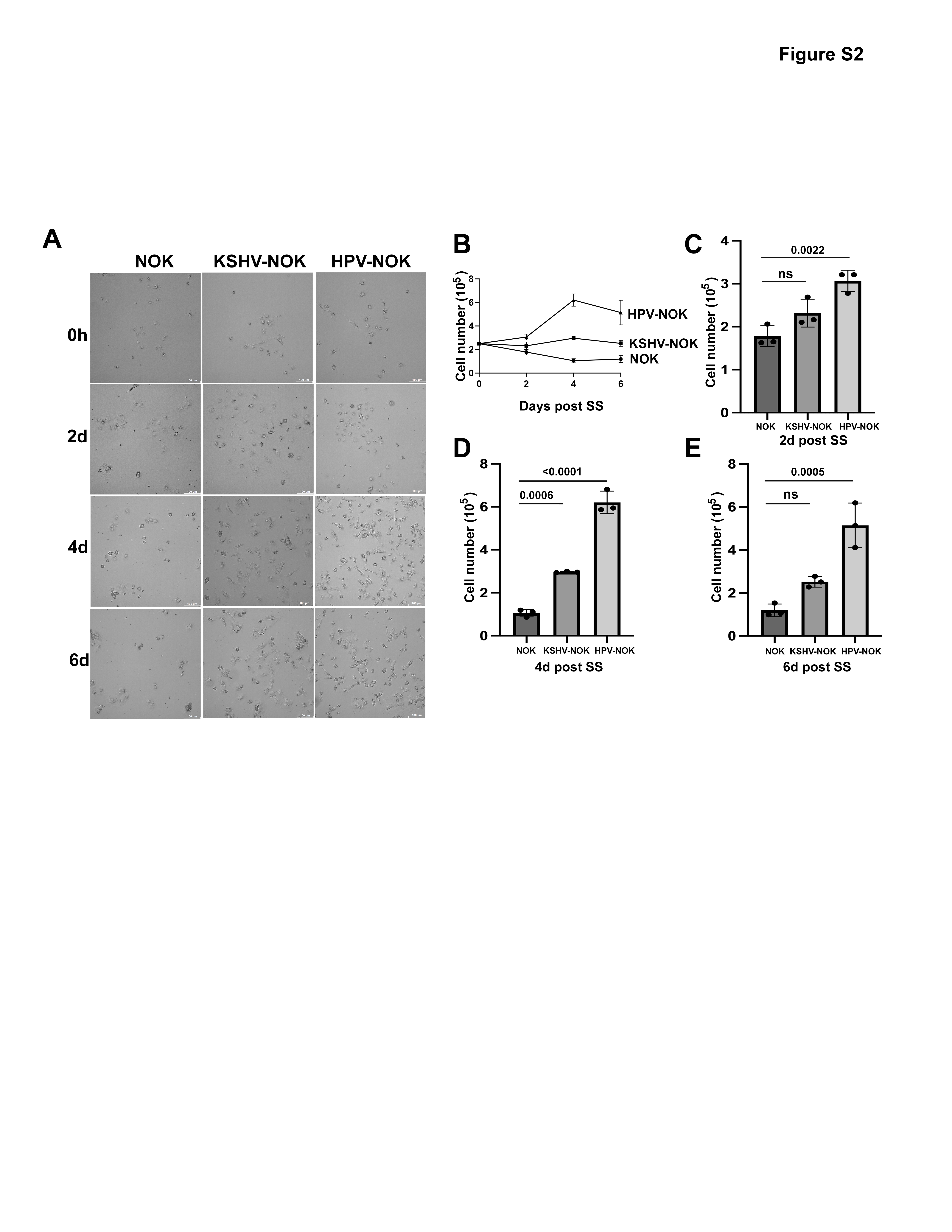

Supplement: Figure S2 — KSHV-NOK and HPV-NOK grow faster than NOK in serum-free keratinocyte basal medium. [file mbio.00484-25-s0002.tif]

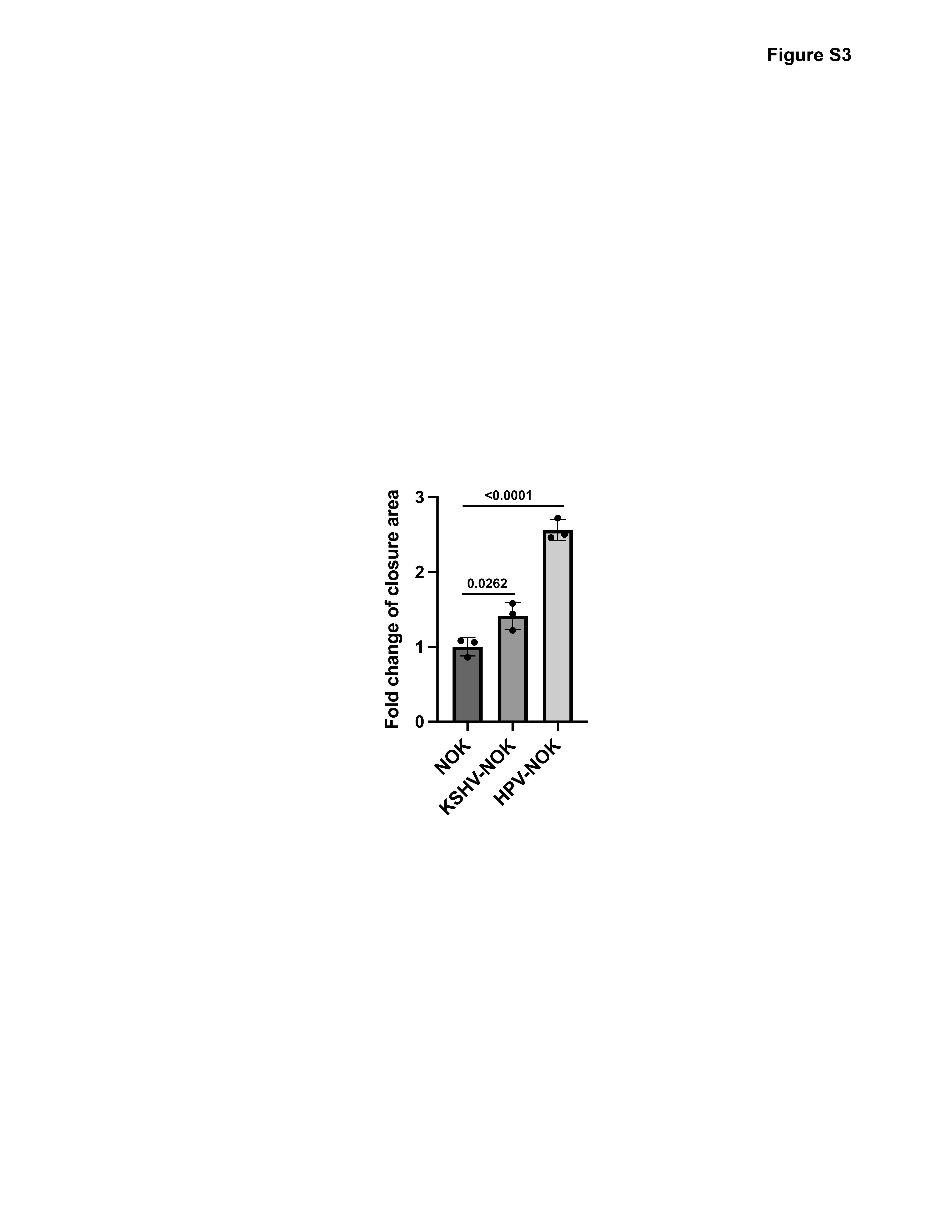

Supplement: Figure S3 — KSHV-NOK and HPV-NOK promote wound healing. [file mbio.00484-25-s0003.tif]

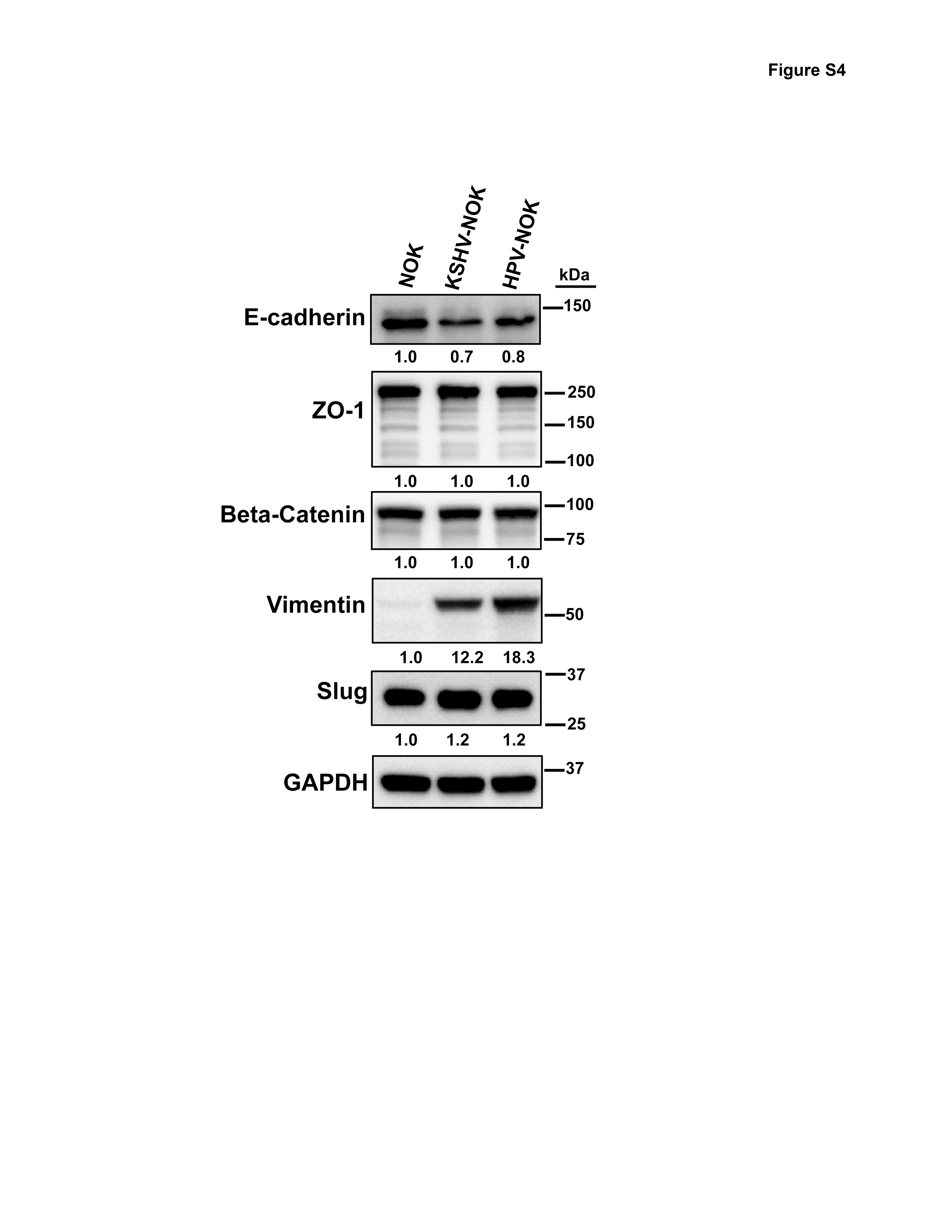

Supplement: Figure S4 — Screening of EMT markers in NOK, KSHV-NOK, and HPV-NOK. [file mbio.00484-25-s0004.tif]

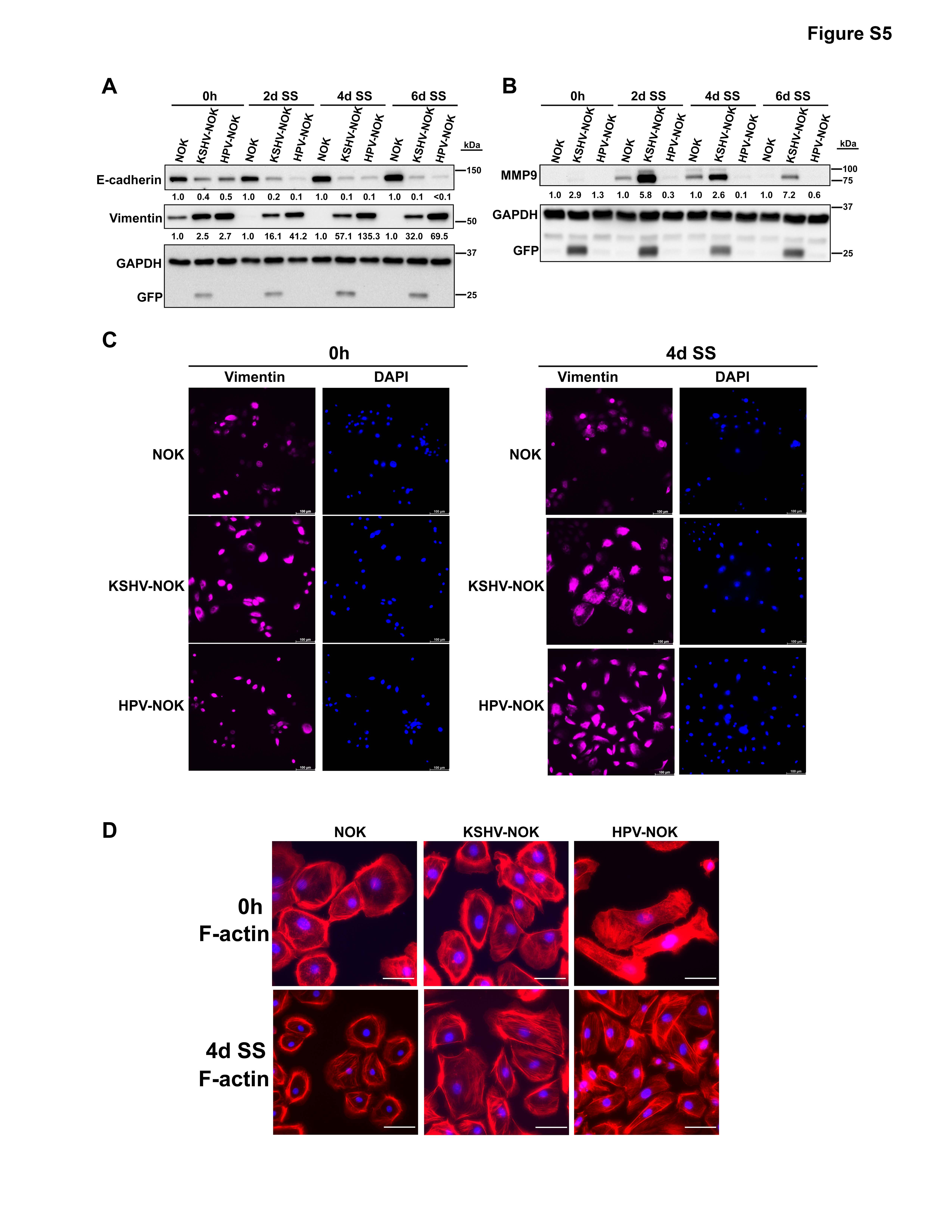

Supplement: Figure S5 — KSHV-NOK and HPV-NOK induce EMT in serum-free keratinocyte basal medium culture environment. [file mbio.00484-25-s0005.tif]

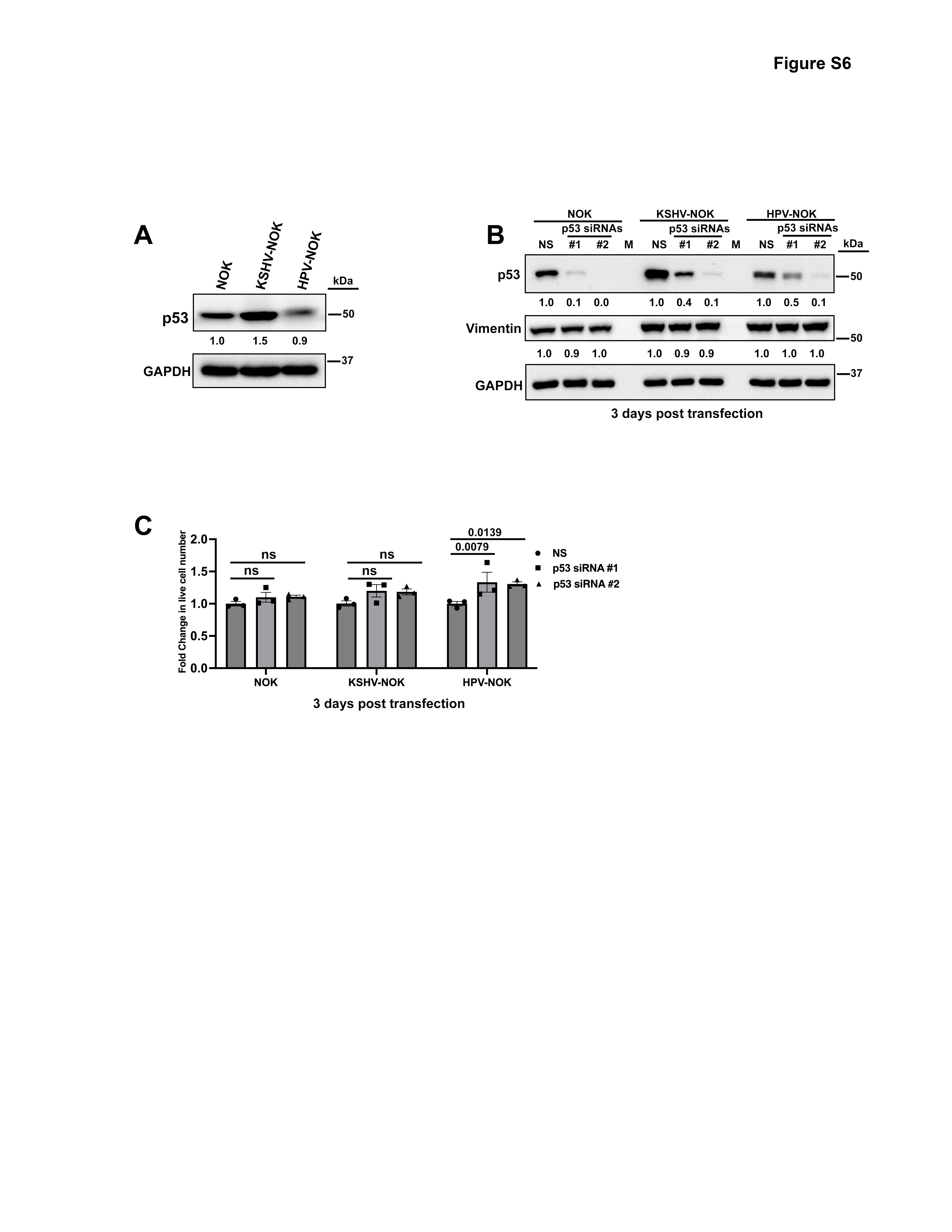

Supplement: Figure S6 — Effect of p53 on NOK, KSHV-NOK, and HPV-NOK cell growth. [file mbio.00484-25-s0006.tif]

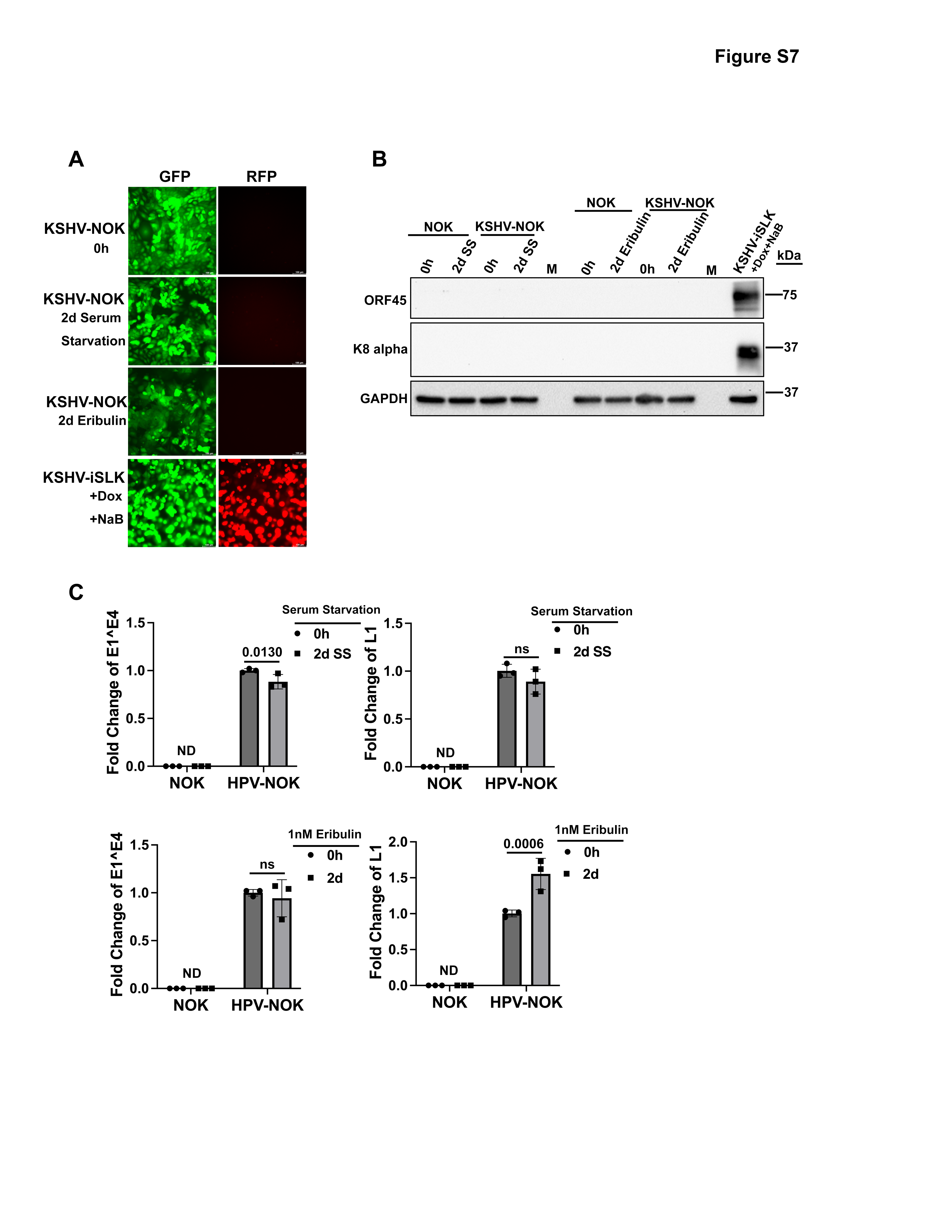

Supplement: Figure S7 — Measurement of KSHV-NOK and HPV-NOK lytic gene expression. [file mbio.00484-25-s0007.tif]
