# Supplementary material for: KSHV and HPV modulate epithelial-to-mesenchymal transition in oral epithelial cells
Source: mBio. 2025 Aug 15;16(9):e00484-25. doi: 10.1128/mbio.00484-25 (PMC12421897; doi:10.1128/mbio.00484-25)
Supplement: Legends — Supplemental figure legends. [file mbio.00484-25-s0008.docx]

**Supplemental Figure Legends**

**Fig. S1. KSHV and HPV31 modulate NOK morphology in serum-free keratinocyte basal medium.** NOK, KSHV-NOK, and HPV-NOK cells were seeded on plates and cultured in E media containing 5% FBS and EGF overnight. Cells were then cultured with serum-free keratinocyte basal media for another 4 days prior to crystal violet staining. Images shown were taken using the 20x objective of a Leica DMi8 fluorescent microscope. **(A)** Shown are representative images for three independent experiments. Scale bar, 50 μm. **(B)** Cell circularity and **(C)** cell roundness of approximately 400 to 1200 cells were measured using ImageJ software. Values represent the average of three independent experiments and error bars represent means ± SEM. p values were analyzed using one-way ANOVA with Dunnett multiple comparisons.

**Fig. S2. KSHV-NOK and HPV-NOK grow faster than NOK in serum-free keratinocyte basal medium.** NOK, KSHV-NOK, and HPV-NOK cells were seeded on plates and cultured in E media containing 5% FBS and EGF overnight. Cells were then cultured with serum-free keratinocyte basal media for an additional 2, 4, and 6 days. **(A)** Representative brightfield images taken with the 20x objective of a Leica DMi8 fluorescent microscope are shown, scale bar, 100μm. **(B-E)** Live cells were counted at different timepoints after trypan blue staining. Live cell numbers were used to generate a growth curve and analyzed separately at each timepoint. Average value represents the mean ± SD of four replicates from an independent experiment, p values were analyzed using one-way ANOVA with Dunnett multiple comparisons. **“**SS” indicates serum starvation.

**Fig. S3. KSHV-NOK and HPV-NOK promote wound healing.** Wound healing 2D migration assays were performed by seeding NOK, KSHV-NOK and HPV-NOK cells into 35mm 2-well culture-insert dishes. After attachment, cells were treated with 10μg/ml of mitomycin C for 3h, and a cell-free gap (wound) was then created and wound healing (i.e. cell migration) was measured at 10h. Brightfield images were taken with the 5x objective of a Leica DMi8 fluorescent microscopy. The wound closure area was measured using ImageJ software. Average value represents the mean ± SD of three replicates. p values were analyzed using one-way ANOVA with Dunnett multiple comparisons.

**Fig. S4. Screening of EMT markers in NOK, KSHV-NOK and HPV-NOK.** NOK, KSHV-NOK, and HPV-NOK cells were seeded in 10cm dishes and cultured in E media containing 5% FBS and EGF for 3 days. Cells were harvested and cell lysates were probed for EMT markers by Western blot. Data shows representative images of three independent experiments.

**Fig. S5. KSHV-NOK and HPV-NOK induce EMT in serum-free keratinocyte basal medium culture environment. (A-B)** NOK, KSHV-NOK, and HPV-NOK cells were cultured in either E media containing 5% FBS and EGF or serum-free keratinocyte basal media for 2, 4, and 6 days. EMT markers (A) and ECM (extracellular matrix) markers (B) were analyzed by Western blot. **(C-D)** NOK, KSHV-NOK, and HPV-NOK cells were cultured in either E media containing 5% FBS and EGF or serum-free keratinocyte basal media for 4 days. Immunofluorescence analysis of vimentin (C) and F-actin staining (D) was performed. Vimentin images were taken with the 20x objective while F-actin images were taken with the 40x objective of a Leica DMi8 fluorescent microscope. Scale bar for Vimentin is 100μm and for F-actin is 50μm. Representative images of three independent experiments (A), two independent experiments (B, D) and one independent experiment (C) are shown. **“**SS” indicates serum starvation.

**Fig. S6. Effect of p53 on NOK, KSHV-NOK and HPV-NOK cell growth. (A)** NOK, KSHV-NOK, and HPV-NOK cells were seeded in 10cm dishes and cultured in E media containing 5% FBS and EGF for 3 days. Cell pellets were harvested for Western blot to measure p53 expression at the protein level. Data shows representative images of three independent experiments. **(B)** Expression levels of p53 and Vimentin were examined by Western blot in p53 siRNA #1- and #2-transfected NOK, KSHV-NOK and HPV-NOK cells at 3 days post transfection, with non-silencing (NS) siRNA performed as a control. Data shows the result of two independent experiments. **(C)** The effect of p53 knockdown on NOK, KSHV-NOK and HPV-NOK cell growth was performed using trypan blue staining at 3 days post transfection. Data represents the average of triplicate from an independent experiment and error bars represent means ± SD, p values were analyzed using two-way ANOVA with Dunnett multiple comparisons.

**Fig. S7. Measurement of KSHV-NOK and HPV-NOK lytic gene expression. (A)** For serum starvation conditions, KSHV-NOK cells were cultured in either E media containing 5% FBS and EGF or serum-free and EGF-free E media for 2 days. For Eribulin treatment, KSHV-NOK cells were cultured in E media containing either DMSO or 1nM Eribulin for 2 days. As a positive control, KSHV-iSLK were lytically induced using 1μg/ml Dox and 1mM Sodium Butyrate for 1 day. GFP and RFP images were taken using 20x objective of a Leica DMi8 fluorescent microscope. Scale bar, 100 μm. Images shown are representative images for three replicates of an independent experiment. **(B)** NOK and KSHV-NOK cell pellets were harvested at 2 days post serum starvation or Eribulin treatment. Lysates were subjected to western blot to measure KSHV lytic genes expression. As a positive control, KSHV-iSLK cells were treated with 1μg/ml Dox and 1mM Sodium Butyrate to induce lytic reactivation. “M” indicates protein Marker. **(C)** For serum starvation conditions, NOK and HPV-NOK cells were cultured in either E media containing 5% FBS and EGF or serum-free and EGF-free E media for 2 days. For Eribulin treatment, NOK and HPV-NOK cells were cultured in E media containing either DMSO or 1nM Eribulin for 2 days. Cell pellets were harvested for RT-qPCR to measure E1^E4 and L1 mRNA level. Average value represents the mean ± SD of three replicates. p values were analyzed using two-way ANOVA with Sidak multiple comparisons. **“**SS” indicates serum starvation.
